# Effect of NAT2, GSTM1 and CYP2E1 genetic polymorphisms on plasma concentration of isoniazid and its metabolites in patients with tuberculosis, and the assessment of exposure-response relationships

**DOI:** 10.3389/fphar.2024.1332752

**Published:** 2024-03-22

**Authors:** Viktorija Ulanova, Agnija Kivrane, Anda Viksna, Leonora Pahirko, Lauma Freimane, Darja Sadovska, Iveta Ozere, Andra Cirule, Eduards Sevostjanovs, Solveiga Grinberga, Dace Bandere, Renate Ranka

**Affiliations:** ^1^ Laboratory of Molecular Microbiology, Latvian Biomedical Research and Study Centre, Riga, Latvia; ^2^ Pharmacogenetics Laboratory, Department of Pharmaceutical Chemistry, Riga Stradins University, Riga, Latvia; ^3^ Centre of Tuberculosis and Lung Diseases, Riga East University Hospital, Upeslejas, Latvia; ^4^ Faculty of Physics, Mathematics, and Optometry, University of Latvia, Riga, Latvia; ^5^ Latvian Institute of Organic Synthesis, Riga, Latvia

**Keywords:** NAT2, GSTM1, CYP2E1, pharmacogenomics, isoniazid, tuberculosis

## Abstract

**Objectives:** Isoniazid is a key drug in the chemotherapy of tuberculosis (TB), however, interindividual variability in pharmacokinetic parameters and drug plasma levels may affect drug responses including drug induced hepatotoxicity. The current study investigated the relationships between isoniazid exposure and isoniazid metabolism-related genetic factors in the context of occurrence of drug induced hepatotoxicity and TB treatment outcomes.

**Methods:** Demographic characteristics and clinical information were collected in a prospective TB cohort study in Latvia (*N* = 34). Time to sputum culture conversion (tSCC) was used as a treatment response marker. Blood plasma concentrations of isoniazid (INH) and its metabolites acetylisoniazid (AcINH) and isonicotinic acid (INA) were determined at three time points (pre-dose (0 h), 2 h and 6 h after drug intake) using liquid chromatography-tandem mass spectrometry. Genetic variations of three key INH-metabolizing enzymes (NAT2, CYP2E1, and GSTM1) were investigated by application PCR- and Next-generation sequencing-based methods. Depending on variables, group comparisons were performed by Student’s *t*-test, one-way ANOVA, Mann-Whitney-Wilcoxon, and Kruskal-Wallis tests. Pearson correlation coefficient was calculated for the pairs of normally distributed variables; model with rank transformations were used for non-normally distributed variables. Time-to-event analysis was performed to analyze the tSCC data. The cumulative probability of tSCC was obtained using Kaplan-Meier estimators. Cox proportional hazards models were fitted to estimate hazard rate ratios of successful tSCC.

**Results:** High TB treatment success rate (94.1%) was achieved despite the variability in INH exposure. Clinical and demographic factors were not associated with either tSCC, hepatotoxicity, or INH pharmacokinetics parameters. Correlations between plasma concentrations of INH and its metabolites were NAT2 phenotype-dependent, while *GSTM1* genetic variants did not showed any effects. CYP2E1*6 (T > A) allelic variant was associated with INH pharmacokinetic parameters. Decreased level of AcINH was associated with hepatotoxicity, while decreased values of INA/INH and AcINH/INH were associated with month two sputum culture positivity.

**Conclusion:** Our findings suggest that CYP2E1, but not GSTM1, significantly affects the INH pharmacokinetics along with NAT2. AcINH plasma level could serve as a biomarker for INH-related hepatotoxicity, and the inclusion of INH metabolite screening in TB therapeutic drug monitoring could be beneficial in clinical studies for determination of optimal dosing strategies.

## 1 Introduction

Tuberculosis (TB) is one of the leading infectious causes of death worldwide. The World Health Organization estimates that 10.6 million people developed TB in 2021 (World Health Organization, 2022). Treatment success rate of 85% for drug-susceptible TB is achieved with 2-month short-course chemotherapy containing isoniazid (INH), rifampicin (RIF), ethambutol (EMB), and pyrazinamide (PZA), followed by INH and RIF for additional 4 months (World Health Organization, 2020). INH is a key drug in the chemotherapy of TB, however, interindividual variability in pharmacokinetic parameters and drug plasma levels may affect drug responses (Ellard and Gammon, 1976; Aarnoutse, 2011). Therapeutic drug monitoring, which utilizes a technique using plasma drug concentration measurements in clinical specimens, was highlighted as a personized treatment approach for the evaluation of the effectiveness of anti-TB treatment and achieving therapeutic goals (Alsultan and Peloquin, 2014; Perumal et al., 2020). However, the current data correlating INH plasma concentrations, adverse drug events and TB treatment outcome are still limited, and the involvement of contributing factors is not sufficiently studied.

Several enzymes are known to metabolize INH, and a detailed model of candidate enzymes in the metabolism of INH in liver cells is described elsewhere (Klein et al., 2016). The main metabolic pathway of INH is acetylation by arylamine N-acetyltransferase 2 (NAT2) to N-acetylisoniazid (AcINH) (Ellard and Gammon, 1976; Daly, 2021). Other INH metabolites, such as hydrazine (Hz), isonicotinic acid (INA), acetylhydrazine (AcHz), and diacetylhydrazine (diAcHz) are generated by hydrolysis, glycine conjugation, hydrazone formation, and further acetylation. None of the INH metabolites are active, while Hz and AcHz are considered to be hepatotoxic (Becker et al., 2007). In turn, INH, AcHz, and Hz are likely oxidized, in part, by cytochrome P450 2E1 (CYP2E1) enzyme into potentially hepatotoxic intermediates, which could be further conjugated by glutathione S-transferase (GST) enzyme family with glutathione and effectively removed by excretion (Klein et al., 2016). Currently, *NAT2* is the most extensively studied pharmacogenetic risk factor for the development of INH-related drug induced hepatotoxicity (DIH) (Daly, 2021). It is well-known that the NAT2 enzyme activity exhibits substantial interindividual variability resulting in different INH plasma half-lives (Ellard and Gammon, 1976; Aarnoutse, 2011). Genetic polymorphisms of *NAT2* gene are responsible for the variations in the acetylation capacity, and the recent meta-analysis study has shown that NAT2 deficiency was significantly associated with the likelihood of experiencing DIH (Richardson et al., 2019). Also, it was proposed that INH dose adjustment based on the *NAT2* genotype before prescribing TB treatment could improve clinical outcome and reduce the risk of INH-induced liver injury, however, the current level of evidence is not sufficient to recommend dose optimization (Azuma et al., 2013; McIlleron and Chirehwa, 2018; Khan and Das, 2022). A number of risk factors for the development of INH-related liver injury have been identified, including age and some environmental factors, such as alcohol consumption, and genetic variations in other drug metabolizing enzymes, such as *CYP2E1* and *GSTM1*, could also be related to the alterations of the INH metabolism and accumulation of toxic substances, which, subsequently, can lead to adverse drug events and affect treatment outcome (Ellard and Gammon, 1976; Yang et al., 2019).

This study has two objectives: (1) to examine the impact of exposure to INH and its metabolites on TB treatment outcome and DIH; (2) to investigate factors which influence variability in INH pharmacokinetics. Towards these objectives we performed pharmacokinetic profiling of INH and its major metabolites (AcINH, INA) in patients with pulmonary TB, and investigated genetic variations of three key INH-metabolizing enzymes (NAT2, CYP2E1, and GSTM1) along with an array of patient-related factors. The obtained data provide a better insight into INH pharmacogenetics thus contributing to future implementation of personalized approach for enhancing the efficacy, tolerability and limiting the toxicity of existing anti-TB medication.

## 2 Materials and methods

### 2.1 Study participants and data collection

Study cohort was comprised of 34 patients with diagnosed drug susceptible pulmonary TB admitted to the Riga East University Hospital (REUH), Centre of Tuberculosis and Lung Diseases. In order to minimize the impact of other health conditions on the study results, the following exclusion criteria were applied: age ≤ 18 years, pregnancy or lactation, history of cancer, other infectious diseases (e.g., HVC, HVB, HIV, and AIDS), and renal failure.

The diagnosis of TB was confirmed radiologically, symptomatically, and/or bacteriologically on Löwenstein–Jensen (L-J) solid medium. Xpert/RIF Ultra test was used for detection of TB and resistance to RIF with additional Line probe assay for resistance-conferring mutation/INH resistance detection before phenotypic drug susceptibility testing. Sputum smear/culture findings, laboratory test values and treatment outcomes were recorded. Time to sputum culture conversion (tSCC) was determined by the number of days to the first negative inoculation of Löwenstein-Jensen (L-J) medium (including the last day), indicating the treatment outcome. “Treatment success” was defined as the sum of “cured” and “treatment completed” (World Health Organization, 2022). All patients received the WHO-recommended treatment regimen for drug-susceptible TB which consistent of a 2-month chemotherapy containing isoniazid INH (4–6 mg/kg), RIF (8–12 mg/kg), PZA (20–30 mg/kg) and EMB (15–25 mg/kg) followed by INH and RIF for additional 4 months. The anti-TB drugs were all single drug products rather than fixed-dose combinations, administered once daily.

Clinical biochemistry tests of liver and renal function were performed at baseline and on day 10–12 of treatment. The reference ranges for transaminases [alanine aminotransferase (ALAT), aspartate aminotransferase (ASAT)], direct and total bilirubin were adopted from specific reference ranges of REUH Laboratory Service laboratory “Gaiļezers” to determine the upper limit of normal range (ULN); the reference range were as follows: alanine aminotransferase (ALAT), 8–41 U/L; aspartate aminotransferase (ASAT), 8–40 U/L; direct (conjugated) bilirubin, < 3.4 µ mol/L; total bilirubin, 5–21 µ mol/L. The DIH case was confirmed based on the increase of ALAT/ASAT values above the ULN on day 10–12 of TB treatment with/without increase of total and conjugated bilirubin levels: mild hyperfermentemia was characterized by elevated serum ALAT/ASAT levels 1.5–5 times above the ULN; moderate hyperfermentemia: 5–10 times above the ULN, and severe hyperfermentemia: >10 times above ULN.

Patient data [age, biological sex, height, weight, sputum microscopy results, time to sputum culture conversion (tSCC), laboratory findings, treatment outcome] were obtained from medical records; smoking status, self-reported alcohol intake were obtained from patients’ questionnaires. Patient’s nutritional status was reported as body mass index (BMI), which was calculated based on height and body weight. According to the TB treatment guidelines, TB treatment was initiated following the directly observed treatment (DOT) strategy for health facility-based patients, and the video observed directly observed treatment (VDOT) strategy was implemented for outpatient department patients.

Patients were enrolled in the study after providing written informed consent for the use of their phenotypic and genetic data. The study was approved by the Central Medical Ethics Committee of Latvia (approval No. 01-29.1/1 and approval No. 01-29.1.2/1736), the Ethics Committee of the Riga East University Hospital (approval No 24-A/15 and approval No. A1/1.1–07.1/20/1809), and the Riga Stradins University Research Ethics Committee (approval No. 6-3/1/6 and approval No. 105/28.01.2016.). This study was conducted in accordance with the Declaration of Helsinki.

### 2.2 DNA and blood plasma samples

Blood sample collection was carried on 10–12 days after the treatment onset. Blood samples were collected by medical personnel using vacutainers with EDTA at three time points: pre-dose (0 h), 2 h, and 6 h after drug intake. Blood samples were immediately centrifuged at 4,000 rpm (3,488 × g) for 15 min at 4°C to separate the plasma; plasma aliquots were immediately frozen and stored at −70°C until further analysis. Genomic DNA was extracted from the peripheral white blood cells using the standard phenol-chloroform method described elsewhere (Sambrook et al., 1989). DNA sample concentrations were measured using a spectrophotometer (ND-1000 UV-VIS Spectrophotometer, NanoDrop Technologies, United States). DNA samples were stored at −20°C, until it was used as a template for PCR amplification.

### 2.3 *NAT2* genotyping

A 1211-bp fragment, which contains the entire coding region of *NAT2* was amplified by PCR using primers P100 5′-GTC​ACA​CGA​GGA​AAT​CAA​ATG​C and P56 5′-GTT​TTC​TAG​CAT​GAA​TCA​CTC​TGC-3’ (Cascorbi et al., 1996). The PCR was performed in a final volume of 25 μL. The master mix contained (per reaction): 1x Phusion Buffer with 7.5 mM MgCl_2_, 200 mM of each dNTPs, 0.3 mM of each primer, 0.4 U of Phusion Hot Start II High-Fidelity DNA polymerase (Thermo Fisher Scientific, United States) and 5 ng of DNA template. The PCR assays were performed under the following conditions: an initial denaturation at 98°C 30 s; 40 cycles of denaturation at 98°C for 10s, primer annealing at 65°C for 30 s, and elongation at 72°C for 30 s; and a final elongation step at 72°C for 5 min. PCR products were purified using the ExoI and FastAP enzymes (Thermo Fisher Scientific, United States) and subsequently sequenced on both DNA strands using P100 and three additional internal primers (P90, 5′-ACA​CAA​GGG​TTT​ATT​TTG​TTC​C-3’; NAT2_R_for, 5′ TAA​TTC​TAG​AGG​CTG​CCA​CAT​C-3’; NAT2_S_for, 5′-GAA​TAC​ATA​CCT​GCA​GAC​GTC​TC-3′) (Cascorbi et al., 1996; Igumnova et al., 2016). Sequencing was performed using the ABI PRISM BigDye Terminator v3.1 Cycle Sequencing Kit (Applied Biosystems) according to the manufacturer’s recommendations on an ABI Prism 3,100 Genetic Analyzer (Perkin-Elmer, United States).

For *NAT2* gene analysis of heterozygous samples, subsequent cloning of 1211-bp PCR fragment into the pJET1.2/blunt cloning vector was performed using the CloneJET PCR Cloning Kit (Thermo Fisher Scientific, United States) according to the manufacturer’s instructions. Plasmids with confirmed *NAT2* gene inserts were purified from bacterial cultures using GeneJET Plasmid Miniprep Kit (Thermo Fisher Scientific, United States), and the inserts were sequenced.

Sequencing data analysis was performed using CodonCode Aligner software (CodonCode Corporation, United States) with the sequence of human gene for arylamine N-acetyltransferase (EC 2.3.1.5) (GenBank: X14672.1) as the reference. A possible NAT2 phenotype was assigned based on the obtained genotyping data of conventional 7-SNP signature, as the presence/absence of signature mutations were linked to the resultant protein activity (Sim et al., 2014). In addition, a database of human *NAT2* alleles was used (http://nat.mbg.duth.gr/Human%20NAT2%20alleles_2013.htm). According to (Stanley and Sim, 2008) individuals were classified as rapid (carrying two rapid *NAT2* alleles), intermediate (one rapid and one slow allele) or slow (two slow alleles) acetylators.

### 2.4 *GSTM1* genotyping

The *GSTM1* gene double deletion genotype (0/0) was detected based on the comparative duplex PCR assay as described previously (Igumnova et al., 2016). Briefly, the PCR master mix contained (per reaction) 1X Taq Buffer with (NH_4_)_2_SO_4_, 2.5 mM MgCl_2_, 100 mM of each dNTPs, 0.2 mM of each primer Beta1 (5′-GGT​TGG​CCA​ATC​TAC​TCC​CAG​G-3′), Beta2 (5′-GCT​CAC​TCA​GTG​TGG​CAA​AG-3′), M1 (5′-CTG​CCC​TAC​TTG​ATT​GAT​GGG-3′) and M2 (5′-CTG​GAT​TGT​AGC​AGA​TCA​TGC-3′), 1.25 U of Taq DNA polymerase and 28 ng of DNA template. PCR assays were performed under the following conditions: an initial denaturation at 95°C for 10 min; 35 cycles of denaturation at 95°C for 30 s, primer annealing at 64°C for 30 s, and elongation at 72°C for 40 s; and a final elongation step at 72°C for 2 min. The PCR products were separated and visualized using electrophoresis on a 1.5% agarose gel containing 0.2 mg of ethidium bromide/ml with transillumination under UV light. The homozygous GSTM1 null genotype (0/0) was detected by the absence of the 273-bp. Individuals who were presented with the 273-bp fragment were classified as GSTM1 plus genotype (A/A or A/0) and contained at least one wild-type allele.

### 2.5 Full-length *CYP2E1* gene analysis

For the full-length *CYP2E1* gene analysis the next-generation sequencing (NGS)-based assay targeting all 9 gene exons with interleaving introns, exonic, untranslated (UTR), upstream and intergenic regions was used. The full-length *CYP2E1* gene sequencing assay, including PCR amplification and NGS paired-end libraries preparation workflow was carried out according to the published protocol (Igumnova et al., 2022) with a few minor modifications in the sequencing data analysis. Specifically, functional annotation of the identified single nucleotide polymorphisms (SNPs) and Indels was performed using Snpeff eff (vGalaxy Version 4.3 + T.galaxy1) and online-based wANNOVAR tool (http://wannovar.wglab.org/) (Wang et al., 2010). Reference SNP reports accumulated in dbSNP database (https://www.ncbi.nlm.nih.gov/snp/), Varsome (https://varsome.com/), PharmGKB (https://www.pharmgkb.org/) and the American College of Medical Genetics and Genomics (ACMG) were used for the detected variant identification and annotation (Whirl-Carrillo et al., 2012).

Sequencing data analysis was performed on the Galaxy online-based platform using the public server at https://usegalaxy.org. (Jalili et al., 2020). Trimmomatic (v0.38) was used to trim adapter sequences and low quality read ends (Phred quality score < 20). Reads were mapped against the human reference genome (GRCh38.p13, GCF 000001405.39) using Map with BWA-MEM (v0.7.17.1). BAM filter (v0.5.9) was applied to keep only mapped reads, discard reads shorter than 50 bp, but Mark duplicates (v2.18.2.2) was used to finalize deduplication. Samtools depth (v1.9) was used to compute the depth at each position in genomic coordinates Chr10 (NC_000010.11, 133524920–133539123). FreeBayes (v1.3.1) for genomic variant calling using BED file in specific target region (NC_000010.11, 133524920–133539123) was implemented. Detected variants were filtered using the following arguments: minimum coverage to process a site ≥10, minimum mapping quality ≥10, minimum base quality ≥20, minimum alternate fracture 0.2, minimum alternate count of observations ≥6 supporting an alternate allele within a single individual in order to evaluate the position. Variants with quality <5 were filtered out to reduce false positive calls. All detected genomic variants were visually inspected using Integrative Genome Viewer, v2.8.9 (Robinson et al., 2011). The sequence analysis and SNV identification were performed using FinchTV and MEGA software (Erasmus, 2021; Taiwo et al., 2022) with the sequence of human *CYP2E1* gene (E.C.1.14.13.n7) (GenBank: NG_008383.1) as the reference.

### 2.6 Liquid chromatography-tandem mass spectrometry (LC-MS/MS)

Blood plasma concentrations of INH and its main metabolites AcINH and INA were determined at three time points (pre-dose (0 h), 2 h and 6 h after drug intake) using liquid chromatography-tandem mass spectrometry (LC-MS/MS) method as previously described (Kivrane et al., 2021). Briefly, plasma **s**amples were analysed using An Acquity UPLC H-Class chromatographic system (Waters, Milford, MA, United States) coupled to a XEVO TQ-S tandem mass spectrometer (Waters, Milford, MA, United States). The chromatographic separation was achieved on the Waters Acquity UPLC BEH C8 column (2.1 mm × 75 mm; 1.7 μm). The multiple reaction monitoring mode (MRM) was used for detection and quantification of the analytes. Data acquisition and analysis were performed using MassLynx software and TargetLynx module (version 4.1., Waters, Milford, MA, United States). Calibration ranges were 0.16–10.0 μg/mL for INH, AcINH and INA, respectively; the lower limits of quantification (LLOQ) were 0.16 μg/mL for INH and both two primary metabolites. The within- and between-run accuracy in all concentration levels ranged from 87.2% to 113.6%, but within- and between-run precision was between 1.6% and 14.9% (at the LLOQ level CV<20%).

### 2.7 Determination of INH pharmacokinetic parameters

The following INH pharmacokinetic parameters were evaluated: (1) INH plasma peak concentration at 2 h (C_max_ 2 h); (2) area under the time–concentration curve 0–6 h (AUC_0–6h_) of INH and its two major metabolites (AcINH and INA); (3) metabolic ratio (MR) AcINH/INH and MR INA/INH (calculated as the ratio of metabolite to the parent drug in 2 h plasma and expressed as a range) (Chen et al., 2017; Sekaggya-Wiltshire, Lamorde, et al., 2018). Therapeutic range for INH was defined as 3–6 μg/mL approximately 2 h after ingestion (Märtson et al., 2021).

### 2.8 Statistical analysis

All statistical analysis were conducted using R statistical software version 4.2.1. (R Core Team (2022), R Foundation for Statistical Computing, Vienna, Austria; https://r-project.org).

Categorical variables were summarized as frequencies and percentages; equality of proportions and association between two-categorical variables were tested using Chi-square tests. When Chi-square test were not applicable, Fisher’s exact test was used instead for association test. Shapiro-Wilk test was used to verify the normality assumption, and, where possible, log-transformation was used to achieve the normal distribution in the data. For normally distributed variables, two or more group comparisons were performed by Student’s *t*-test and one-way ANOVA, respectively. Models with rank transformations were used for non-normally distributed variables, two and three or more group comparisons were performed by Mann-Whitney-Wilcoxon and Kruskal-Wallis test, respectively.

Pearson correlation coefficient with the corresponding 95% confidence interval and *p*-values were calculated for the pairs of normally distributed variables; model with rank transformations were used for non-normally distributed variables (i.e., AcINH/INH and INA/INH ratios). When applicable, during the correlation analysis, the six pharmacokinetic parameters of INH were adjusted for age, sex, and INH dose (mg/kg); AcINH and INA (AUC0-6 h) values were log-transformed and the correlations were also stratified by NAT2 acetylator status and *GSTM1* genotypes.

Time-to-event analysis was performed to analyse the tSCC data. Patients whose treatment outcomes were “treatment completed” or “lost to follow-up” were considered as censored. The cumulative probability of tSCC, stratified by *GSTM1* genotypes and *NAT2* phenotypes, was obtained using Kaplan-Meier estimators. Log-rank tests were performed to assess differences between the groups. Median tSCC were calculated for both GSTM1 and NAT2 groups. Additionally, the mean tSCC, restricted at time point 60 days, was compared between subgroups of *GSTM1* and *NAT2* (R library survRM2). Confidence intervals were constructed for the group medians and for the differences of restricted mean tSCC. Cox proportional hazards models were fitted to estimate hazard rate (HR) ratios of successful tSCC. In a case of proportional hazards, the HR of successful tSCC can be interpreted as the odds for an individual in the group with higher hazard to reach the endpoint first (Spruance et al., 2004). In the Cox regression models the following predictors were included for the whole study cohort (*n* = 34): (1) age, sex, smear microscopy, and standardized values of AcINH/INH ratio; (2) age, sex, smear microscopy, and standardized values of INA/INH ratio; (3) *GSTM1* genotype, NAT2 phenotype, CYP2E1*6 (T > A) allelic variant (rs6413432), and sputum smear microscopy.

Linkage disequilibrium (LD) was assessed by Ensembl Linkage Disequilibrium Calculator (https://www.ensembl.org/Homo_sapiens/Tools/LD?db=core) utilizing genotyping data from the 1000 Genomes Project database for SNP pairwise comparisons through the r^2^ coefficient. All the SNPs were filtered using a pairwise tagging algorithm with high LD r^2^ measure ≥ 0.8 and minor allele frequency (MAF) > 0.05. All detected SNPs genotypes were tested for agreement with Hardy-Weinberg equilibrium (HWE) by Fisher exact probability test based on the genotype distributions in this study. As a result, seven SNPs were chosen as the representatives to analyse the association with patients’ clinical characteristics. *p*-values ≤ 0.05 were considered statistically significant.

## 3 Results

### 3.1 TB treatment outcome and DIH cases

Patients’ characteristics are presented in Table 1 and Supplementary Table S1. In total, 34 patients with pulmonary TB were included. All patients were Caucasians. The mean patient age was 46.3 (±12.2) years, and the majority of the patients were male (82.4%, 28/34), current smokers (73.5%, 25/34), and with normal BMI (61.8%, 21/34) (Table 1). One-third of the patients (32.4%, 11/34) reported daily alcohol intake. In 55.9% (19/34) cases smear microscopy result was positive.

**TABLE 1:** Study participants’ characteristics (patients with pulmonary tuberculosis, *n* = 34).

Characteristic		
Categorical data. no/total (%)		
Biological sex	Male	28 (82.4)
	Female	6 (17.7)
Smoking status	Non-smoker	9 (26.5)
	Smoker	25 (73.5)
BMI*	Underweight	8 (23.5)
	Normal	21 (61.8)
	Overweight	5 (14.7)
Smear microscopy	Negative	15 (44.1)
	Positive	19 (55.9)
Self-reported alcohol intake	Yes	11 (32.4)
	No	23 (67.7)
Non-categorical data. mean (±SD)		
Age		46.3 (±12.2)
INH dose (mg/kg)		4.64 (±0.83)

*Body mass index groups: underweight, <18.5 kg/m^2^; normal, 18.5–24.9 kg/m^2^; overweight, 25.0–29.9 kg/m^2^.

For categorical variables, data are presented as frequencies (%). For continuous normally distributed variables, data are presented as means (± SD). BMI, body mass index; INH, isoniazid; AcINH, N-acetylisoniazid; INA, isonicotinic acid; AUC, area under the concentration-time curve; C_max_, maximum concentration; MR, metabolic ratio; NAT2, N-acetyltransferase 2; SA, slow acetylator; IA, intermediate acetylator; GSTM1, glutathione S-transferase mu 1 class; SD, standard deviation; IQR, interquartile range; OR, odds ratio.

TB treatment outcome was successful for all but two patients (treatment success rate = 94.1%): the majority of the patients were cured (28/34, 82.4%), and four (11.8%) patients completed the treatment (Table 2). Two patients (5.9%) were lost to follow-up. Overall, tSCC data were available for 28 patients (i.e., those with “cured” treatment outcome). Median tSCC was 52 (11–197) days; 16 patients (57.1%) became *M. tuberculosis* culture-negative in less than 60 days, while for 12 patients (42.9%) tSCC was > 60 days.

**TABLE 2:** Treatment outcome, response and hepatotoxicity in patients with TB (*n* = 34) depending on various factors.

Characteristics		Treatment outcome*		tSCC (n = 28**)		Hepatotoxicity	
		Cured	Treatment completed	<60 days	>60 days	Yes	No
All patients. no/total (%)		28/34 (82.4)	4/34 (11.8)	16/28 (57.1)	12/28 (42.9)	4/34 (11.8)	30/34 (88.2)
Categorical data. no/total (%)							
Biological sex	Male	24/28 (85.7)	3/28 (10.7)	13/24 (54.2)	11/24 (45.8)	2/28 (7.1)	26/28 (92.9)
	Female	4/6 (66.7)	1/6 (16.7)	3/4 (75.0)	1/4 (25.0)	2/6 (33.3)	4/6 (66.7)
	*p*-value	0.51		0.44		0.071	
	OR (95% CI)	2.0 (0.16, 24.3)		0.39 (0.04, 4.35)		0.15 (0.02, 1.42)	
Smoking status	Non-smoker	5/9 (55.6)	3/9 (33.3)	3/5 (60.0)	2/5 (40.0)	2/9 (22.2)	7/9 (77.8)
	Smoker	23/25 (92.0)	1/25 (4.0)	13/23 (56.5)	10/23 (43.5)	2/25 (8.0)	23/25 (92.0)
	*p*-value	**0.039**		0.89		0.26	
	OR (95% CI)	13.8 (1.2, 161.7)		0.87 (0.12, 6.2)		0.30 (0.04, 2.6)	
BMI***	Underweight	5/8 (62.5)	2/8 (25.0)	3/5 (60.0)	2/5 (40%)	1/8 (12.5)	7/8 (87.5)
	Normal	19/21 (90.5)	1/21 (4.8)	10/19 (52.6)	9/19 (47.4)	2/21 (9.5)	19/21 (90.5)
	Overweight	4/5 (80.0)	1/5 (20.0)	3/4 (75.0)	1/4 (25%)	1/5 (20.0)	4/5 (80.0)
	*p*-value	0.21		0.71		0.81	
Smear microscopy	Negative	9/15 (60.0)	4/15 (26.7)	7/9 (77.8)	2/9 (22.2)	2/15 (13.3)	13/15 (86.7)
	Positive	19/19 (100.0)	0/19 (0.0)	9/19 (47.4)	10/19 (52.6)	2/19 (10.5)	17/19 (89.5)
	*p*-value	**0.02**		0.13		0.8	
	OR (95% CI)			0.26 (0.04, 1.57)		0.76 (0.09, 6.2)	
Daily alcohol intake	Yes	10/11 (90.9)	1/11 (9.1)	6/10 (60.0)	4/10 (40.0)	2/11 (18.1)	9/11 (81.8)
	No	18/23 (78.3)	3/23 (13.0)	10/18 (55.6)	8/18 (44.4)	2/23 (8.7)	21/23 (91.3)
	*p*-value	1		0.82		0.42	
	OR (95% CI)	1.6 (0.15, 18.2)		1.2 (0.25, 5.8)		2.33 (0.28, 19.2)	
Non-categorical data. mean (±SD)							
Age		48.1 (±10.8)	43.0 (±17.1)	47.4 (±12.2)	48.9 (±9.1)	52.5 (±9.0)	45.5 (±12.5)
	*p*-value	0.6		0.72		0.24	
INH dose (mg/kg)		4.67 (±0.74)	4.24 (±1.41)	4.68 (±0.78)	4.66 (±0.71)	4.93 (±0.91)	4.60 (±0.82)
	*p*-value	0.58		0.94		0.53	
AUC _0–6h_, µg/mL·h	INH	10.8 (±5.5)	12.7 (±6.8)	10.1 (±5.9)	11.8 (±5.1)	13.8 (±4.7)	10.9 (±5.6)
	*p*-value	0.63		0.4		0.31	
	AcINH	7.5 (±4.0)	4.42 (±1.7)	8.6 (±4.0)	6.1 (±3.7)	4.6 (±0.7)	7.4 (±3.9)
	*p*-value	0.088		0.102		**0.019**	
	INA	2.1 (±0.9)	1.5 (±0.5)	2.3 (±1.0)	1.9 (±0.8)	1.7 (±0.6)	2.1 (±0.9)
	*p*-value	0.18		0.2		0.43	
C_max_ 2h, µg/mL	INH	2.6 (±1.5)	3.0 (±1.6)	2.5 (±1.5)	2.8 (±1.5)	2.7 (±1.5)	2.7 (±1.5)
	*p*-value	0.67		0.51		0.99	
Non-categorical data. median (IQR)							
Metabolic ratio	AcINH/INH	0.46 (0.35–1.64)	0.27 (0.20–1.36)	0.91 (0.42–1.74)	0.39 (0.33–0.50)	0.40 (0.32–0.44)	0.47 (0.34–1.66)
	*p*-value	0.12		0.053		0.26	
	INA/INH	0.21 (0.13–0.28)	0.12 (0.08–0.31)	0.23 (0.16–0.31)	0.14 (0.10–0.21)	0.12 (0.10–0.15)	0.19 (0.12–0.28)
	*p*-value	0.19		**0.026**		0.12	

*Two patients were lost to follow-up.

**Sputum culture conversion was confirmed in 28 patients.

***Body mass index groups: underweight, <18.5 kg/m^2^; normal, 18.5–24.9 kg/m^2^; overweight, 25.0–29.9 kg/m^2^.

For categorical variables, data are presented as frequencies (%), equality of proportions between groups was tested using Chi-square test (or Fisher's exact test where Chi-square was not applicable) and is reported using p-values. Odds ratio (95% CI) was calculated. For continuous normally distributed variables, data are presented as means (± SD), comparison of means was performed by Student's t-test or one-way ANOVA between two or three groups, respectively; AcINH and INA was log-transformed before running the tests. Models with rank tranformations were used for non-normally distributed variables (AcINH/INH and INA/INH), and data are presented as medians (IQR); the comparison of medians was performed by Mann-Whitney-Wilcoxon or Kruskal-Wallis test between two or three groups, respectively. *p*-values ≤ 0.05 are highlighted in bold.BMI, body mass index; tSCC, time to sputum culture conversion; INH, isoniazid; AcINH, N-acetylisoniazid; INA, isonicotinic acid; AUC, area under the concentration-time curve; C_max_, maximum concentration; MR, metabolic ratio; NAT2, N-acetyltransferase 2; SA, slow acetylator; IA, intermediate acetylator; GSTM1, glutathione S-transferase mu 1 class; SD, standard deviation; IQR, interquartile range; OR, odds ratio.

In total, after 10–12 days of therapy, transaminase levels increased in 4 patients (11.76%): mild and moderate DIH developed in one patient each, and severe DIH—in two patients. In two of these patients ALAT/ASAT levels were increased before therapy onset, and in 1 patient simultaneous changes in total and conjugated bilirubin levels were recorded (Table 2, Supplementary Table S1; Supplementary Figure S1).

### 3.2 INH pharmacokinetic data

Mean plasma values of INH and both metabolites are presented in Table 3. Overall, 64.7% (22/34) of the patients had INH C_max_ 2 h value below the therapeutic range window (<3.0 μg/mL; Supplementary Table S1); the mean INH C_max_ 2 h of the cohort was 2.7 (±1.4) µg/mL.

**TABLE 3:** Pharmacokinetic parameters for isoniazid stratified by patient characteristics.

*Total*		Pharmacokinetic parameters AUC_0–6h_, µg/mL·h; mean (±SD)			C_max_, 2 h, µg/mL; mean (±SD)	Metabolic ratio; median (IQR)	
		INH	AcINH	INA	INH	AcINH/INH	INA/INH
		11.2 (±5.5)	7.0 (±3.8)	2.0 (±0.9)	2.7 (±1.4)	0.46 (0.34–1.56)	0.16 (0.12–0.28)
Biological sex	Male (*n* = 28)	11.2 (±5.5)	7.4 (±4.0)	2.1 (±0.9)	2.8 (±1.5)	0.46 (0.34–1.64)	0.19 (0.13–0.28)
	Female (*n* = 6)	11.3 (±5.9)	5.5 (±2.2)	1.6 (±0.6)	2.5 (±1.5)	0.45 (0.35–0.49)	0.11 (0.10–0.20)
	*p*-value	0.98	0.28	0.22	0.66	0.84	0.26
Smoking status	Non-smoker (*n* = 9)	11.2 (±5.9)	6.7 (±3.2)	1.7 (±0.6)	2.6 (±1.4)	0.43 (0.35–1.70)	0.15 (0.10–0.22)
	Smoker (*n* = 25)	11.3 (±5.4)	7.2 (±4.1)	2.1 (±1.0)	2.8 (±1.5)	0.46 (0.33–1.34)	0.20 (0.12–0.28)
	*p*-value	0.99	0.91	0.19	0.69	0.79	0.36
BMI*	Underweight (*n* = 8)	11.8 (±4.1)	7.8 (±4.0)	2.3 (±1.3)	2.9 (±0.9)	0.47 (0.40–1.32)	0.21 (0.13–0.27)
	Normal (*n* = 21)	11.7 (±6.2)	7.0 (±4.2)	1.9 (±0.8)	2.9 (±1.7)	0.36 (0.32–1.34)	0.15 (0.11–0.24)
	Overweight (*n* = 5)	8.6 (±3.9)	6.2 (±1.9)	1.8 (±0.6)	1.9 (±0.9)	0.49 (0.43–1.66)	0.23 (0.17–0.36)
	*p*-value	0.51	0.79	0.62	0.42	0.65	0.62
Daily alcohol intake	Yes (*n* = 11)	11.1 (±5.0)	6.7 (±4.1)	1.9 (±0.7)	2.7 (±1.4)	0.46 (0.35–1.0)	0.20 (0.14–0.27)
	No (*n* = 23)	11.3 (±5.8)	7.2 (±3.8)	2.3 (±1.2)	2.8 (±1.5)	0.46 (0.34–1.67)	0.16 (0.10–0.26)
	*p*-value	0.91	0.54	0.49	0.8	0.72	0.42

For normally distributed variables, data are presented as means (± SD); AcINH and INA AUC_0-6h_ values were log-transformed before running the tests. For normally distributed variables, comparison of means was performed by Student's t-test or one-way ANOVA between two or three groups, respectively. Models with rank tranformations were used for non-normally distributed variables (i.e. AcINH/INH and INA/INH ratios), and data are presented as medians (IQR). Comparison of medians was performed by Mann-Whitney-Wilcoxon or Kruskal-Wallis test between two or three groups, respectively.INH, isoniazid; AcINH, N-acetylisoniazid; INA, isonicotinic acid; AUC, area under the concentration-time curve; C_max_, maximum concentration; BMI, body mass index; SD, standard deviation; IQR, interquartile range.

*Underweight <18.5 kg/m^2^; normal 18.5–24.9 kg/m^2^; overweight 25.0–29.9 kg/m^2^.

### 3.3 Genetic variability of INH-metabolizing enzymes

#### 3.3.1 *NAT2* and *GSTM1*

During the genotyping analysis, *NAT2* SA phenotype-related genotypes were detected in 67.7% (23/34), and *NAT2* IA—in 32.4% (11/34) of the patients, respectively (Table 4). *GSTM1*-null genotype was present in 58.8% (20/34), and *GSTM1*-plus genotype—in 41.2% (14/34) of the patients, respectively.

**TABLE 4:** Pharmacokinetic parameters for isoniazid stratified by *NAT2* phenotype, *GSTM1* genotype, and *CYP2E1* SNP (rs) variants.

Total		AUC_0–6h_, µg/mL·h; EMM, SE, CI 95%			Cmax, 2 h, µg/mL	Metabolic ratio; median (IQR)	
		INH	AcINH	INA	INH	AcINH/INH	INA/INH
		11.3, SE = 1.26, CI=(8.7, 13.9)	5.7, SE = 1.14, CI=(4.39, 7.39)	1.69, SE = 1.1, CI=(1.39, 2.06)	2.64, SE = 0.33, CI=(1.97, 3.31)	0.46 (0.34–1.56)	0.16 (0.12–0.28)
*NAT2* phenotype	SA (*n* = 23)	13.22, SE = 1.07, CI=(11.03, 15.40)	4.48, SE = 1.09, CI=(3.78, 5.37)	1.59, SE = 1.11, CI=(1.29, 1.96)	3.01, SE = 0.32, CI=(2.35, 3.66)	0.35 (0.31–0.46)	0.13 (0.10–0.16)
	IA (*n* = 11)	5.87, SE = 1.54, CI=(2.72, 9.02)	11.02, SE = 1.13, CI=(8.58, 14.30)	2.02, SE = 1.16, CI=(1.50, 2.74)	1.62, SE = 0.46, CI=(0.68, 2.56)	1.71 (1.65–2.11)	0.38 (0.28–0.43)
	*p*-value	**<0.001**	**<0.001**	0.13	**0.007**	**<0.001**	**<0.001**
*GSTM1* genotype	null (*n* = 20)	11.2, SE = 1.59, CI=(7.94, 14.5)	5.64, SE = 1.17, CI=(4.06, 7.85)	1.87, SE = 1.10, CI=(1.54, 2.28)	2.68, SE = 0.41, CI=(1.84, 3.52)	0.46 (0.35–1.64)	0.18 (0.13–0.30)
	plus (*n* = 14)	11.4, SE = 1.69, CI=(7.91, 14.8)	5.75, SE = 1.192, CI=(4.06, 8.17)	1.78, SE = 1.12, CI=(1.40, 2.24)	2.60, SE = 0.44, CI=(1.70, 3.49)	0.42 (0.34–1.16)	0.15 (0.11–0.26)
	*p*-value	0.93	0.93	0.72	0.87	0.77	0.74
*CYP2E1* SNP ID	Presence						
rs6413432	No (*n* = 30)	10.4, SE = 1.27, CI= (7.85, 13)	6.05, SE = 1.14, CI=(4.62, 7.92)	1.77, SE = 1.11, CI=(1.43, 2.17)	2.43, SE = 0.33, CI= (1.76, 3.11)	0.468 (0.353–1.658)	0.207 (0.132–0.281)
	Yes (*n* = 4)	16.4, SE = 2.73, CI= (10.78, 22)	3.97, SE = 1.33, CI=(2.21, 7.10)	1.32, SE = 1.24, CI=(0.85, 2.06)	3.91, SE = 0.712, CI= (2.46, 5.37)	0.275 (0.212–0.335)	0.084 (0.078–0.091)
	*p*-value	**0.047**	0.166	0.214	0.057	**0.011**	**0.003**
rs2515641	No (*n* = 25)	10.9, SE = 1.52, CI= (7.76, 14.0)	5.75, SE = 1.17, CI=(4.18, 7.85)	1.68, SE = 1.12, CI=(1.32, 2.13)	2.52, SE = 0.393, CI= (1.71, 3.32)	0.457 (0.338–1.665)	0.204 (0.12–0.282)
	Yes (*n* = 9)	12.0, SE = 1.98, CI= (7.98, 16.1)	5.64, SE = 1.22, CI=(3.74, 8.5)	1.71, SE = 1.16, CI=(1.26, 2.34)	2.87, SE = 0.514, CI= (1.82, 3.92)	0.428 (0.348–0.487)	0.159 (0.114–0.211)
	*p*-value	0.619	0.928	0.91	0.558	0.673	0.748
rs41299398	No (*n* = 25)	10.8, SE = 1.42, CI = 7.92, 13.7)	5.47, SE = 1.15, CI=(4.06, 7.32)	1.56, SE = 1.11, CI=(1.26, 1.93)	2.53, SE = 0.368, CI= (1.78, 3.28)	0.428 (0.334–1.636)	0.148 (0.105–0.279)
	Yes (*n* = 9)	12.5, SE = 2.06, CI= (8.25, 16.7)	6.36, SE = 1.23, CI=(4.18, 9.78)	2.10, SE = 1.16, CI=(1.54, 2.85)	2.93, SE = 0.534, CI= (1.84, 4.02)	0.479 (0.424–0.604)	0.217 (0.165–0.263)
	*p*-value	0.472	0.502	0.08	0.501	0.41	0.275
rs915908	No (*n* = 22)	10.1, SE = 1.39, CI= (7.26, 13.0)	5.7, SE = 1.16, CI=(4.22, 7.69)	1.60, SE = 1.12, CI=(1.28, 2.01)	2.33, SE = 0.360, CI= (1.6, 3.07)	0.468 (0.348–1.611)	0.182 (0.122–0.268)
	Yes (*n* = 12)	13.5, SE = 1.78, CI= (9.91, 17.2)	5.7, SE = 1.21, CI=(3.9, 8.41)	1.88, SE = 1.15, CI=(1.41, 2.5)	3.24, SE = 0.459, CI= (2.3, 4.18)	0.423 (0.307–0.888)	0.152 (0.104–0.292)
	*p*-value	0.09	0.991	0.321	0.085	0.245	0.701
rs8192766	No (*n* = 30)	10.9, SE = 1.33, CI= (8.20, 13.6)	5.87, SE = 1.14, CI=(4.44, 7.69)	1.69, SE = 1.11, CI=(1.37, 2.09)	2.51, SE = 0.339, CI= (1.81, 3.21)	0.455 (0.341–1.658)	0.162 (0.115–0.281)
	Yes (*n* = 4)	13.8, SE = 3.01, CI= (7.64, 20.0)	4.76, SE = 1.36, CI=(2.55, 8.94)	1.70, SE = 1.26, CI=(1.06, 2.74)	3.54, SE = 0.770, CI= (1.97, 5.12)	0.352 (0.211–0.67)	0.178 (0.129–0.216)
	*p*-value	0.365	0.522	0.978	0.206	0.278	0.396
rs41299422	No (*n* = 30)	11.0, SE = 1.27, CI= (8.42, 13.6)	5.81, SE = 1.14, CI=(4.45, 7.54)	1.70, SE = 1.10, CI=(1.39, 2.08)	2.58, SE = 0.332, CI= (1.91, 3.26)	0.468 (0.338–1.658)	0.207 (0.116–0.281)
	Yes (*n* = 4)	14.7, SE = 3.09, CI= (8.38, 21.0)	4.53, SE = 1.37, CI=(2.37, 8.58)	1.59, SE = 1.27, CI=(0.97, 2.6)	3.41, SE = 0.805, CI= (1.76, 5.05)	0.385 (0.33–0.43)	0.139 (0.115–0.161)
	*p*-value	0.236	0.423	0.782	0.307	0.222	0.211
rs7081484	No (*n* = 29)	11.7, SE = 1.42, CI= (8.76, 14.5)	5.75, SE = 1.15, CI=(4.26, 7.69)	1.69, SE = 1.12, CI=(1.35, 2.10)	2.69, SE = 0.369, CI= (1.94, 3.45)	0.455 (0.339–1.636)	0.159 (0.105–0.279)
	Yes (*n* = 5)	9.9, SE = 2.60, CI= (4.58, 15.2)	5.58, SE = 1.30, CI=(3.25, 9.58)	1.71, SE = 1.22 CI=(1.14, 2.58)	2.46, SE = 0.677, CI= (1.07, 3.84)	0.487 (0.319–1.219)	0.211 (0.145–0.232)
	*p*-value	0.546	0.93	0.94	0.754	0.993	0.565

For the analysis, six pharmacokinetic parameters of INH were adjusted for age, sex, and dose (mg/kg). For normally distributed variables, data are presented as marginal means; comparison of the means was performed by Student's t-test. AcINH and INA were log-transformed before running the tests. Models with rank tranformations were used for non-normally distributed variables (AcINH/INH and INA/INH ratios), however, the results did not differ from the unadjusted analysis, and therefore are presented as medians (IQR); the comparison of medians was performed by Mann-Whitney-Wilcoxon test between two groups. *p*-values < 0.05 are highlighted in bold. INH, isoniazid; AcINH, N-acetylisoniazid; INA, isonicotinic acid; AUC, area under the concentration-time curve; Cmax, maximum concentration; MR, metabolic ratio; NAT2, N-acetyltransferase 2; SA, slow acetylator; IA, intermediate acetylator; GSTM1, glutathione S-transferase mu 1 class; CYP2E1, cytochrome P450, 2E1 class; SNP, single nucleotide polymorphism; EMM, Estimated Marginal Means; SE, standard error; CI, Confidence Interval; IQR, interquartile range.

#### 3.3.2 Selection of tagging *CYP2E1* SNPs

Sequencing data quality for the study samples set (*n* = 34) after the applied quality filters are summarized in Supplementary Table S2. All detected SNPs were grouped according to the region of location and functional classification. The majority of detected SNPs were intronic (*n* = 60); three were exonic (missense), three variants located in a 3′ untranslated region (3 UTR), five deletion-insertion variants, eight intergenic, one multiple nucleotide variation, and five - upstream SNPs (Supplementary Table S3). All but nine SNPs conformed to HWE (*p* > 0.05). In the sample set, four allelic variants, i.e., CY2E1*1B (rs2070676, G>C), CYP2E1*4 (rs6413419 G>A), CYP2E1*6 (rs6413432 T>A), and CYP2E1*7A (rs2070673 A>T) were identified. Of all identified SNPs, the variants rs2515641 (T>C), CY2E1*1B (G>C) (rs2070676), and CYP2E1*6 (T>A) (rs6413432) were of clinical significance level 3 (Khrunin et al., 2010; Iacobucci et al., 2013; Richardson et al., 2018; Wu et al., 2019; Yu et al., 2019).

When selecting tagging SNPs, priority was given to variants found to be associated with impaired drug metabolism, i.e., with potential effects on CYP2E1 enzyme activity and/or potentially leading to adverse drug reactions, including DIH. Based on the MAF and HWE results, as well as previously reported possible effects on CYP2E1 enzyme activity, 7 SNPs were selected for further analysis: rs8192766, rs6413432, rs2515641, rs41299422, rs7081484, rs41299398, and rs915908.

### 3.4 Association of non-genetic and pharmacokinetic factors with TB treatment outcome and tSCC

No significant associations of age, BMI group, self-reported alcohol intake, or INH dose (mg/kg) with treatment outcome or tSCC were found (Table 2). There was a significant association between smoking status and treatment outcome, where smokers in comparison to non-smokers had 13.8 times higher odds of treatment outcome “cured” in comparison to the “treatment completed” and “lost to follow up” outcomes (*p* = 0.039); however, we did not observe significant associations of smoking status and the overall treatment success (Table 2). Similarly, there was a significant association between sputum microscopy results and treatment outcome: the treatment outcome “cured” could be assigned to 100% of microscopy-positive patients in comparison to the microscopy-negative patients (outcome “cured”: 60.0%, “treatment completed”: 26.7%, “lost to follow up”: 13.3%) (*p* = 0.02). However, again, sputum microscopy result was not associated with the overall treatment success in our cohort. Further analysis indicated that smoking was significantly associated with positive sputum microscopy (*p* = 0.025) (Supplementary Table S4).

Within the INH pharmacokinetic parameters, median INA/INH ratio was significantly higher in patients with tSCC < 60 days in comparison to those with tSCC > 60 days (*p* = 0.026) (Table 2).

**FIGURE 1:**
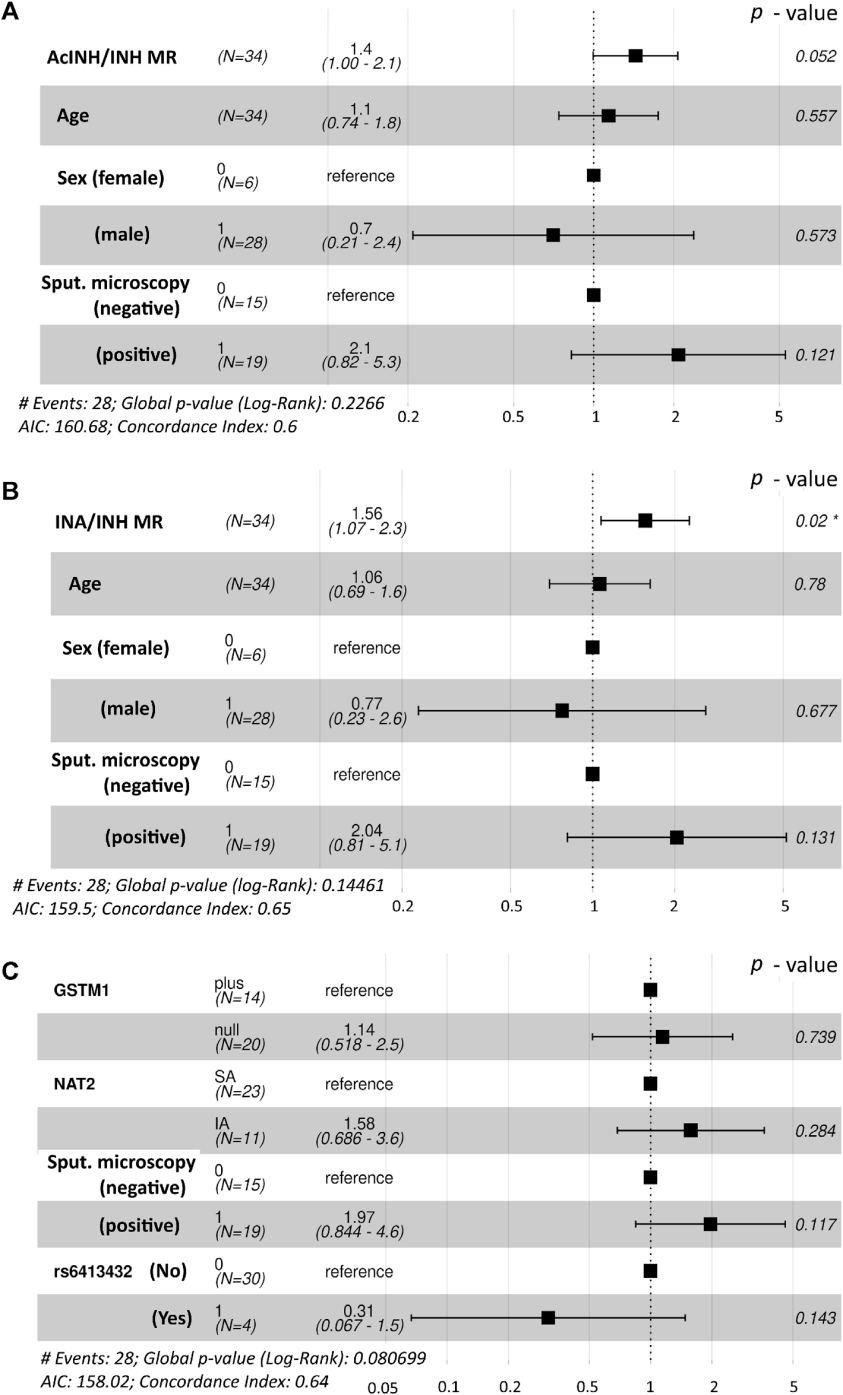
Forest plot demonstrating the hazard rate ratios of successful time to sputum culture conversion obtained from fitted Cox proportional hazards model. Statistically significant *p*-value is marked with asterisk (*). **(A)**. Interactions between age, sex and sputum microscopy results were used along with the AcINH/INH metabolic ratio. **(B)** Interactions between age, sex and sputum microscopy results were used along with the INA/INH metabolic ratio **(C)** Interactions between NAT2 acetylator status, *GSTM1* genotype and the presence of CYP2E1*6 (T>A) allelic variant (rs6413432) were used along with the sputum microscopy results. NAT2, N-acetyltransferase 2; GSTM1, glutathione S-transferase mu 1 class; MR, metabolic ratio; INH, isoniazid; AcINH, N-acetylisoniazid; INA, isonicotinic acid.

Global log-rank *p*-value of the Cox regression model incorporating age, sex, smear microscopy, and standardized values of AcINH/INH ratio was *p* = 0.227 and concordance index was 0.6; the estimated hazard rate (HR) ratios are illustrated (Figure 1A). Based on the preceding information, the following observations were found for the whole cohort: the odds of an individual with a given standardized MR (AcINH/INH) value to reach SCC first compared to an individual with one unit lower standardized MR value were 1.4 (95% CI, 1.00–2.1), *p* = 0.052; a one unit increase of the standardized AcINH/INH ratio value gave HR/(1 + HR) = 58% probability of healing first. There was no significant effect of other predictors on HR of treatment success.

Global log-rank *p*-value of the Cox regression model incorporating age, sex, smear microscopy, and standardized values of INA/INH ratio was *p* = 0.145 and concordance index was 0.65; the estimated hazard rate (HR) ratios are illustrated (Figure 1B). The odds of an individual with a given standardized MR (INA/INH) value to reach SCC first compared to an individual with one unit lower standardized MR value were 1.56 (95% CI, 1.07–2.3), *p* = 0.02; a one unit increase of the standardized INA/INH ratio value gave HR/(1+HR) = 61% probability of healing first.

### 3.5 Association of non-genetic and pharmacokinetic factors with DIH

No significant associations of age, BMI group, self-reported alcohol intake, or INH dose (mg/kg) with hepatotoxicity were found (Table 2). Biological sex was slightly associated with DIH, with males having 85% lower odds of hepatotoxicity than women, however, statistical significance was not reached (*p* = 0.071). Within the INH pharmacokinetic parameters, AcINH AUC0-6 h was significantly lower in patients with DIH (*p* = 0.019).

### 3.6 Association of factors with INH pharmacokinetic parameters

Further, we analysed the possible impact of various non-genetic and genetic factors on the variability in INH pharmacokinetics.

None of the analysed pharmacokinetic parameters differed significantly between patients’ groups when stratified by biological sex, smoking status, BMI, and self-reported daily alcohol intake (Table 2). Also, neither age, nor INH dose (mg/kg) correlated with any of INH pharmacokinetics parameters (Supplementary Figures S2, S3).

**FIGURE 2:**
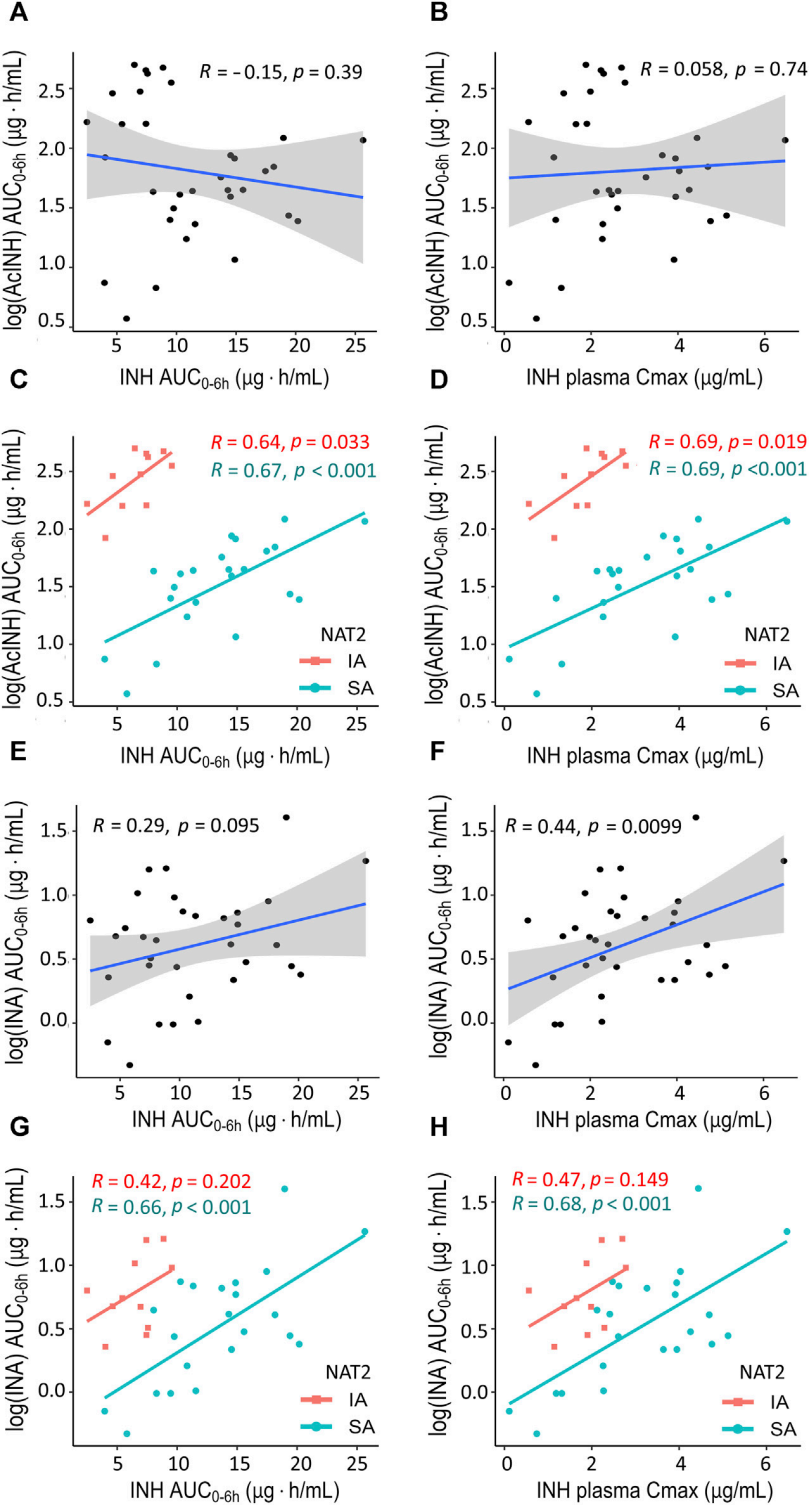
XY plots showing correlation analysis results for isoniazid and two metabolites in blood plasma of patients with tuberculosis. **(A)** Acetylisoniazid (AUC_0–6h_ µg·h/mL) *versus* isoniazid (AUC_0–6h_ µg·h/mL) in the whole study cohort. **(B)** Acetylisoniazid (AUC_0–6h_ µg·h/mL) *versus* isoniazid C_max_ (2 h, µg/mL) in the whole study cohort. **(C)** Acetylisoniazid (AUC_0–6h_ µg·h/mL) *versus* isoniazid (AUC_0–6h_ µg·h/mL), patient samples were stratified based on NAT2 SA and IA acetylator status. **(D)** Acetylisoniazid (AUC_0–6h_ µg·h/mL) *versus* isoniazid C_max_ (2 h, µg/mL), patient samples were stratified based on NAT2 SA and IA acetylator status. **(E)** Isonicotinic acid (AUC_0–6h_ µg·h/mL) *versus* isoniazid (AUC_0–6h_ µg·h/mL) in the whole study cohort. **(F)** Isonicotinic acid (AUC_0–6h_ µg·h/mL) *versus* isoniazid C_max_ (2 h, µg/mL) in the whole study cohort. **(G)** Isonicotinic acid (AUC_0–6h_ µg·h/mL) *versus* isoniazid (AUC_0–6h_ µg·h/mL), patient samples were stratified based on NAT2 SA and IA acetylator status. **(H)** Isonicotinic acid (AUC_0–6h_ µg·h/mL) *versus* isoniazid C_max_ (2 h, µg/mL), patient samples were stratified based on NAT2 SA and IA acetylator status. AcINH and INA AUC_0–6h_ values were log-trans formed before running the tests. Shaded areas represent the 95% confidence interval of the observed concentration mean values. The solid lines represent the Pearson correlation coefficient. Dots represent the observed data for a single patient. INH, isoniazid; AcINH, acetylisoniazid; INA, izonicotinic acid; AUC, area under the concentration-time curve; C_max_, maximum concentration; NAT2, N-acetyltransferase 2; SA, slow acetylator; IA, intermediate acetylator.

In the whole cohort, no correlations were observed between INH (AUC_0–6h_) and either AcINH (AUC_0–6h_) or INA (AUC_0–6h_) (Figures 2A, E); also, no correlation was observed between INH C_max_ and AcINH (AUC_0–6h_) (Figure 2B). However, a significant correlation was observed between INH C_max_ and INA (AUC_0–6h_) (*p* = 0.0099) (Figure 2F).

Correlations between the pharmacokinetic parameters became significantly stronger when the patients were stratified based on the *NAT2* acetylator status; the majority of the observed correlation coefficients were statistically significant, and clear separation between *NAT2* groups was observed (Figures 2C, D, G, H). On the other hand, stratification of the patients based on the *GSTM1* genotype did not improve the correlation values for the pharmacokinetic parameters, and clear group separation was not observed (Supplementary Figure S4).

**FIGURE 3:**
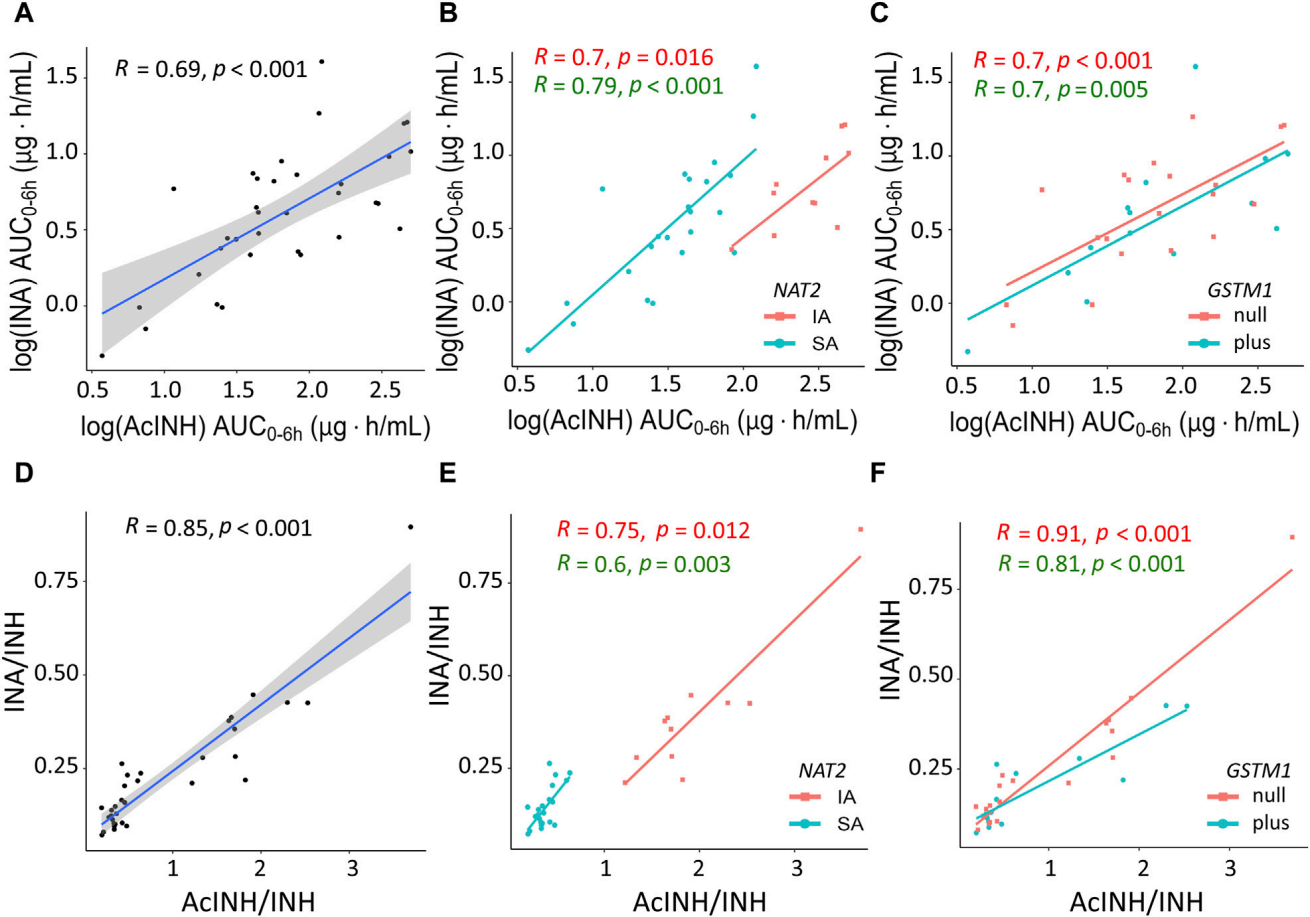
XY plots showing correlation analysis results for isoniazid metabolites and metabolic ratios in blood plasma of patients with tuberculosis. **(A)** Acetylisoniazid (AUC_0–6h_ µg·h/mL) *versus* isonicotinic acid (AUC_0–6h_ µg·h/mL) in the whole study cohort. **(B)** Acetylisoniazid (AUC_0–6h_ µg·h/mL) *versus* isonicotinic acid (AUC_0–6h_ µg·h/mL), patient samples were stratified based on NAT2 SA and IA acetylator status. **(C)** Acetylisoniazid (AUC_0–6h_ µg·h/mL) *versus* isonicotinic acid (AUC_0–6h_ µg·h/mL), patient samples were stratified based on *GSTM1* genotypes. **(D)** Correlation between INA/INH and AcINH/INH ratios in the whole study cohort. **(E)** Correlation between INA/INH and AcINH/INH ratios, patient samples were stratified based on NAT2 SA and IA acetylator status. **(F)** Correlation between INA/INH and AcINH/INH ratios, patient samples were stratified based on *GSTM1* genotypes. The solid lines represent the Pearson correlation coefficient. AcINH and INA (AUC_0–6h_ µg·h/mL) values were log-trans formed before running the tests. Spearman correlation was used for non-normally distributed variables (i.e., AcINH/INH and INA/INH ratios). Shaded areas represent the 95% confidence interval of the observed concentration mean values. Dots represent the observed data for a single patient. INH, isoniazid; AcINH, acetylisoniazid; INA, izonicotinic acid; AUC, area under the concentration-time curve; NAT2, N-acetyltransferase 2; SA, slow acetylator; IA, intermediate acetylator; GSTM1, glutathione S-transferase mu 1 class.

In the whole cohort, significant correlation was observed between both INH metabolites INA (AUC_0–6h_) and AcINH (AUC_0–6h_), as well as between AcINH/INH and INA/INH ratios (Figures 3A, D). Again, group separation was observed for *NAT2* SA and IA acetylators, but not for the *GSTM1* genotypes (Figures 3B, C, E, F).

After adjustment by sex, age and INH dose, the mean and median values of the pharmacokinetics parameters differed significantly between *NAT2* SA and IA groups, except for INA AUC_0–6h_ (Table 4). More specifically, INH AUC_0–6h_ and INH C_max_ values were significantly higher, while AcINH AUC_0–6h_ values were significantly lower in *NAT2* slow acetylators. Also, median values of both AcINH/INH and INA/INH ratios were significantly lower in the *NAT2* SA group. None of pharmacokinetic parameters differed significantly between *GSTM1*-plus and -null genotypes.

Similarly, pharmacokinetic parameters were compared between patient groups divided according to the presence/absence of the each *CYP2E1* SNPs. Overall, only rs6413432 claimed a statistically significant difference for three INH pharmacokinetic parameters: INH AUC_0–6h_, values were significantly higher, while median values of both AcINH/INH MR and INA/INH MR were significantly lower in patients with rs6413432 (Table 4).

### 3.7 Association of genetic factors with treatment outcome, tSCC and DIH

As some INH pharmacokinetic parameters were associated with tSCC and DIH, and the impact of genetic factors on INH pharmacokinetics was observed, we further investigated possible association of genetic factors with treatment outcome, tSCC and DIH.

Based on the univariate analysis, there were no significant associations between treatment outcome and any of genetic factors studied (Table 5). DIH occurred only in the patients with NAT2 SA phenotype (*N* = 4); however, statistically significant difference was not observed between NAT2 SA and IA groups (Table 5). A trend of association between NAT2 phenotypes and tSCC was detected, with SA having 80% lower odds of tSCC within 60 days in comparison to IA (OR = 0.2; 95% CI, 0.03–1.22); however, statistical significance was not reached (*p* = 0.069) (Table 5).

**TABLE 5:** Treatment outcome and hepatotoxicity in patients with TB (*n* = 34) stratified by NAT2 phenotype, *GSTM1* genotype, and *CYP2E1* SNP (rs) variants.

Total			Treatment outcome*		tSCC (*n* = 28**)		Hepatotoxicity	
			Cured	Treatment completed	<60 days	>60 days	Yes	No
*NAT2* phenotype	SA	23/34 (67.7)	18/23 (78.3)	3/23 (13.0)	8/18 (44.4)	10/18 (55.6)	4/23 (17.4)	19/23 (82.6)
	IA	11/34 (32.4)	10/11 (90.9)	1/11 (9.1)	8/10 (80.0)	2/10 (20.0)	0/11 (0.0)	11/11 (100.0)
	*p*-value	0.059	1.0		0.069		0.14	
	OR^1^ (95% CI)	na	0.6 (0.05, 6.6)		0.2 (0.03, 1.22)		na	
*GSTM1* genotype	null	20/34 (58.8)	17/20 (85.0)	2/20 (10.0)	9/17 (52.9)	8/17 (47.1)	2/20 (10.0)	18/20 (90.0)
	plus	14/34 (41.2)	11/14 (78.6)	2/14 (14.3)	7/11 (63.6)	4/11 (36.4)	2/20 (14.3)	12/14 (85.7)
	*p*-value	0.39	0.68		0.58		0.7	
	OR^2^ (95% CI)	na	1.6 (0.19, 12.6)		0.64 (0.14, 3.0)		0.67 (0.08, 5.4)	
*CYP2E1* SNP ID	Presence							
rs6413432	No	30/34 (88.2)	26/30 (86.7)	2/30 (6.7)	16/26 (61.5)	10/26 (38.5)	3/30 (10.0)	27/30 (90.0)
	Yes	4/34 (11.8)	2/4 (50.0)	2/4 (50.0)	0/0 (0.0)	2/2 (100.0)	1/4 (25.0)	3/4 (75.0)
	*p*-value	<0.001	0.106		0.34		0.961	
	OR (95% CI)	na	0.08 (0.01, 0.87)		na		3 (0.23, 38.74)	
rs2515641	No	25/34 (73.5)	20/25 (80.0)	3/25 (12.0)	12/20 (60.0)	8/20 (40.0)	3/25 (12.0)	22/25 (88.0)
	Yes	9/34 (26.5)	8/9 (88.9)	1/9 (11.1)	4/8 (50.0)	4/8 (50.0)	1/9 (11.1)	8/9 (88.9)
	*p*-value	<0.001	1.0		0.952		1.0	
	OR (95% CI)	na	1.2 (0.11, 13.32)		0.67 (0.13, 3.47)		0.92 (0.08, 10.14)	
rs41299398	No	25/34 (73.5)	20/25 (80.0)	4/25 (16.0)	10/20 (50.0)	10/20 (50.0)	3/25 (12.0)	22/25 (88.0)
	Yes	9/34 (26.5)	8/9 (88.8)	0/9 (0.0)	6/8 (75.0)	2/8 (25.0)	1/9 (11.1)	8/9 (88.9)
	*p*-value	<0.001	0.537		0.432		1.0	
	OR (95% CI)	na			3 (0.48, 18.6)		0.92 (0.08, 10.14)	
rs915908	No	22/34 (64.7)	17/22 (77.3)	3/22 (13.6)	11/17 (64.7)	6/17 (35.3)	2/22 (9.1)	20/22 (90.9)
	Yes	12/34 (35.3)	11/12 (91.7)	1/12 (8.3)	5/11 (45.5)	6/11 (54.6)	2/12 (16.7)	10/12 (83.3)
	*p*-value	0.12	1.0		0.539		0.922	
	OR (95% CI)	na	1.94 (0.18, 21.12)		0.45 (0.1, 2.14)		2 (0.24, 16.36)	
rs8192766	No	30/34 (88.2)	25/30 (83.3)	3/30 (10.0)	14/25 (56.0)	11/25 (44.0)	4/30 (13.3)	26/30 (86.7)
	Yes	4/34 (11.8)	3/4 (75.0)	1/4 (25.0)	2/3 (66.7)	1/3 (33.3)	0/4 (0.0)	4/4 (100.0)
	*p*-value	<0.001	1.0		1.0		1.0	
	OR (95% CI)	na	0.36 (0.03, 4.66)		1.57 (0.13, 19.67)		na	
rs41299422	No	30/34 (88.2)	24/30 (80.0)	4/30 (13.3)	15/24 (62.5)	9/24 (37.5)	4/30 (13.3)	26/30 (86.7)
	Yes	4/34 (11.8)	4/4 (100.0)	0/4 (0.0)	1/4 (25.0)	3/4 (75.0)	0/4 (0.0)	4/4 (100.0)
	*p*-value	<0.001	1.0		0.391		1.0	
	OR (95% CI)	na			0.2 (0.02, 2.23)		na	
rs7081484	No	29/34 (85.3)	24/29 (82.8)	3/29 (10.3)	13/24 (54.2)	11/24 (45.8)	4/29 (13.8)	25/29 (86.2)
	Yes	5/34 (14.7)	4/5 (80.0)	1/5 (20.0)	3/4 (75.0)	1/4 (25.0)	0/5 (0.0)	5/5 (100.0)
	*p*-value	<0.001	1.0		0.815		0.894	
	OR (95% CI)	na	0.5 (0.04, 6.08)		2.54 (0.23, 28.02)		na	

*Two patients were lost to follof up and were omitted from calculations.

**Sputum culture conversion was observed in 28 patients.

For categorical variables, data are presented as frequencies (% of the total), equality of proportions between groups was tested using Chi-square test (or Fisher's exact test where Chi-square was not applicable), and is reported using *p*-values. Odds ratio (95% CI) was calculated.

OR^1^ *, NAT2* SA over IA; OR^2^ *, GSTM1* null over plus.

NAT2, N-acetyltransferase 2; SA, slow acetylator; IA, intermediate acetylator; GSTM1, glutathione S-transferase mu 1 class; CYP2E1, cytochrome P450, 2E1 class; SNP, single nucleotide polymorphism. tSCC, time to sputum culture conversion; OR, odds ratio; na, not applicable.

No significant association between *GSTM1* groups and tSCC was detected, and no significant associations were found for any of 7 analysed SNPs of *CYP2E1* gene with either treatment outcome, tSCC, or hepatotoxicity (Table 5).

**FIGURE 4:**
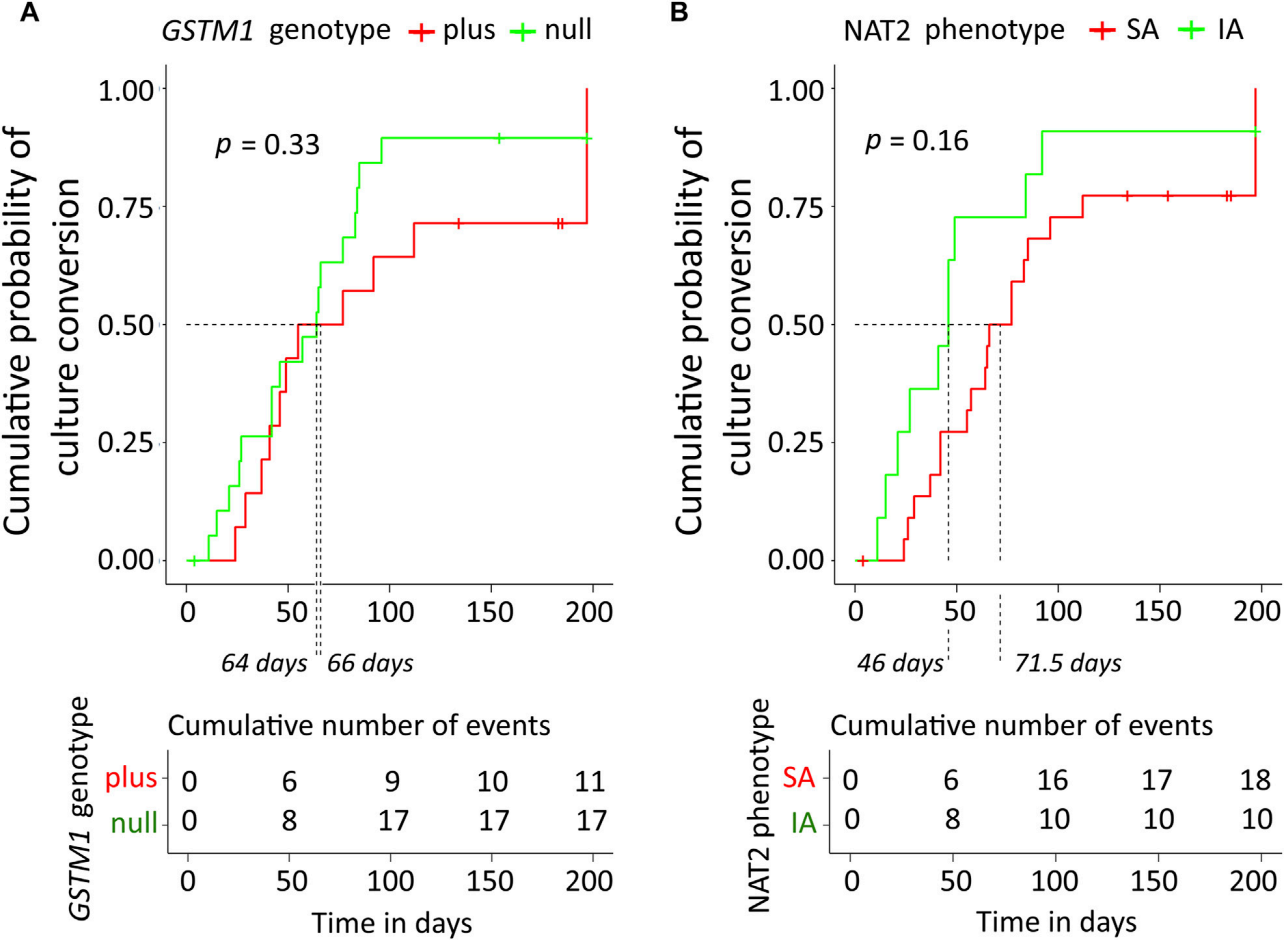
Kaplan-Meier curves of cumulative probability of the sputum culture conversion time along with the table of cumulative number of events. **(A)** Data were stratified based on *GSTM1* genotype. **(B)** Data were stratified based on NAT2 SA and IA acetylator status. The log-rank test was used for comparison of the curves, the corresponding *p*-values are displayed. Median treatment times in subgroups are indicated by dashed lines. NAT2, N-acetyltransferase 2; SA, slow acetylator; IA, intermediate acetylator; GSTM1, glutathione S-transferase mu 1 class.

Cumulative probabilities of the sputum culture conversion event were estimated as the complements of Kaplan-Meier curves (Figure 4). According to the log-rank test, there was no significant difference in tSCC between the genotypes of either NAT2 (*p* = 0.16), or *GSTM1* (*p* = 0.33). Based on the *GSTM1* stratification, the median treatment times were 64 (95% CI, 42–85) and 66 (95% CI, 46–197) days for null vs. plus genotype, respectively. The mean tSCC, restricted at time 60 days, were 46.7 (95% CI, 38.9–54.5) vs. 50 (95% CI, 43.6–56.5) days for null vs. plus genotype, respectively; the difference between plus and null groups was 3.4 days (95% CI, 13.5–6.7), *p* = 0.51. Based on the NAT2 phenotype stratification, the median treatment times were 46 (95% CI, 27–197) and 71.5 (95% CI, 57–112) days for NAT2 IA and SA individuals, respectively. The mean tSCC, restricted at time 60 days, were 39.6 (95% CI, 29.3–49.9) vs. 52.4 (95% CI, 47.2–57.5) days for NAT2 IA and SA individuals, respectively; the difference between NAT2 IA and SA groups was 12.7 days (95% CI, 1.2–24.3), *p* = 0.03 (Figure 4).

However, in the Cox regression model for the whole study cohort (*n* = 34) incorporating *GSTM1* genotype, NAT2 phenotype, CYP2E1*6 (T > A) allelic variant (rs6413432), and smear microscopy, none of the predictors contributed significantly on HR for the respond to treatment (i.e., tSCC); global log-rank *p*-value of the model was *p* = 0.081 and concordance index was 0.64 (Figure 1C).

## 4 Discussion

This is the prospective study aimed to decipher the impact of pharmacokinetic, pharmacogenetic and patient-related factors on the response to TB treatment, TB treatment outcome and the risk of DIH, and to investigate possible links between these variables. For this task, along with the array of patient- and disease-related factors, we evaluated six pharmacokinetic parameters of the parent drug INH and its two metabolites, AcINH and INA, for more accurate assessments of the data obtained (Akkerman et al., 2014; Sturkenboom et al., 2015). In our study, a high TB treatment success rate was achieved (94.1%). However, we did not observe any associations between patient-related factors with either DIH or treatment outcome. Molla and colleagues have reported that males were significantly less likely to develop hepatotoxicity than females (Molla et al., 2021); in our study similar trend was observed, however, statistical significance was not reached (*p* = 0.071). Smoking status was considered to be a baseline risk factor associated with negative impact on the TB treatment success, delaying conversion of the sputum culture, and extending the time of TB treatment (Silva et al., 2018; World Health Organization, 2022). Here, smoking was highly associated with positive sputum microscopy results confirming the negative smoking impact. However, both smokers and patients with positive sputum microscopy results were more likely to achieve treatment outcome “cured” in comparison to the “treatment completed” or “lost to follow up” outcomes (*p* < 0.05). This result could be related to the fact that patients with positive sputum microscopy result were treated in the hospital until the negative sputum microscopy was confirmed, rather than received ambulatory treatment for TB, thus achieving better treatment compliance. These findings could have some implications for TB eradication programmes. NAT2 enzyme plays a key role in biotransformation of xenobiotics including first-line anti-TB drug INH (Mthiyane et al., 2020). The *NAT2* gene is highly polymorphic, and more than 65 variants containing one or more SNPs in the 870 bp long coding region have previously been reported (Hein and Doll, 2012); in turn, the state of NAT2 activity in an individual depends on combinations of rapid and slow alleles (Hein and Doll, 2012; Mthiyane et al., 2020). Worldwide, significant differences in the distribution and frequency of NAT2 alleles between ethnic populations exist; the overall proportions of the acetylator phenotypes in our study cohort was 68% for SA and 32% for IA carriers, similar to those reported in Caucasian populations in Europe and the United States (Sabbagh et al., 2011; Bisso-Machado et al., 2016).

Data from our study confirmed previous reports reviewed in (Hong et al., 2020) showing that INH and its metabolites AcINH and INA blood plasma levels were significantly different between the NAT2 slow and intermediate acetylators: INH AUC_0–6h_ and INH C_max_ were significantly higher, and AUC_0–6h_ values for both metabolites were significantly lower in the SA group in comparison to the IA group; consequently, the median AcINH/INH and INA/INH ratio values were also significantly lower in the SA group. Also, significant NAT2 inter-phenotypic differences in INH and metabolites correlation patterns were detected.

According to the proposed INH metabolism pathway, INH, Hz and AcHz are metabolized by NAT2 enzyme and thus are subjects to the same acetylation polymorphism (Balhara et al., 2021). Three metabolites have been proposed to be responsible for INH-induced liver injury, AcHz, Hz and a metabolite resulting from the bioactivation of INH itself (Metushi et al., 2011). In our study, AcINH AUC_0–6h_ values were significantly lower in patients with DIH suggesting impaired INH acetylation in these cases; in addition, slow acetylation of toxicologically active AcHz to inactive diAcHz could be expected.The frequency of DIH observed in our cohort (11.76%), as well as the association of a slow acetylation profile with DIH, are consistent with the data reported previously (Sistanizad et al., 2011; Perwitasari et al., 2018; Khan et al., 2019). In our small cohort, statistical significance was reached for this INH no other INH pharmacogenetic parameters reached statistical significance, thus these results indicated that AcINH plasma level could serve as a biomarker for DIH.GSTs are important phase II detoxification isozymes that catalyse the conjugation of reduced form of glutathione to toxic metabolites including in the INH metabolic pathway, thereby reducing their reactivity toward cellular macromolecules (Sodhi et al., 1996; Eaton and Bammler, 1999). GST genetic polymorphisms, especially the variants of *GSTM1* gene, have been extensively studied, and the absence of *GSTM1* activity due to null mutation was associated with accumulation of toxic intermediates leading to DIH in some studies (Wang et al., 2016). The proportion of the *GSTM1*-null genotype in our Caucasian cohort was 59%; however, we did not observe any effects of *GSTM1* genotype on DIH, TB treatment outcome, or any of the six INH pharmacokinetic parameters studied.

CYP2E1 is another enzyme involved in the Phase I biotransformation reactions of INH (Cheng et al., 2013). Previous studies on the pharmacogenetic variability of *CYP2E1* have resulted in evidence-based clinical annotations of specific genetic polymorphisms and corresponding allelic variants with consequent different phenotypes in regulatory pathways that effect the pharmacokinetics and treatment response (https://cpicpgx.org/guidelines), but controversial associations of *CYP2E1* genotype with risk of INH-related hepatotoxicity were reported (Lei et al., 2021). In our study, statistically significant associations between CYP2E1*6 (T > A) allelic variant (rs6413432) and three INH pharmacokinetic parameters, i.e., increase in INH AUC_0–6h_, and decrease in both AcINH/INH and INA/INH ratios, were detected; however, no direct proofs for contribution in either DIH or treatment outcome were observed. None of other detected SNPs, including two allelic variants CYP2E1*4 (rs6413419) and CYP2E1*7A (rs2070673) which were previously associated with potential effects on CYP2E1 enzymatic activity, showed any statistically significant associations.

CYP2E1*6 allelic variant, recognized by *Dra* I restriction enzyme, is located in intron 6 of the gene (Fang et al., 2017). Previously, it was suggested that this polymorphism is associated with the *CYP2E1* gene expression at mRNA level (Uematsu et al., 1994). Our data indicate that CYP2E1*6 was related to the decreased amounts of AcINH and INA; however, the mechanism of interaction between *CYP2E1* genetic polymorphisms and INH plasma levels is unclear. Clinical studies have reported the interactions of INH with CY2E1 substrates including observations of interphenotypic difference for INH-related inhibition of *CYP2E1* activity, which was more extensive in slow acetylators (O’Shea et al., 1997). However, *in vitro* studies did not observe any significant inhibition of CYP2E1 by INH or its metabolites (Balhara et al., 2021). Also, treatment of drug-susceptible TB involves several drugs, including RIF, which is a CYP2E1 inducer, thus drug-drug interactions could potentially interfere with INH metabolism or manifestation of INH-related adverse drug reactions through increased production of reactive metabolites, especially in slow acetylators (Shen et al., 2008; Yamada et al., 2009). Additional studies are required to determine the possible functional implications of the *CYP2E1* SNVs by assessing the drug response and clinical manifestations of the identified variants.

Another objective of our study was to determine the possible relationship between INH pharmacokinetic data and TB treatment outcome. First, it should be noted that, in our study, INH C_max_ at 2 h time point was below the therapeutic reference range in the majority of the patients (64.7%). Considering the overall good treatment success rates, our findings support the opinion that the current therapeutic ranges are probably set too high (Perumal et al., 2020). Further, our results showed that INA/INH ratios were significantly higher in patients with SCC time less than 60 days. This assertion was further supported by the findings showing that a one unit increase of the standardized AcINH/INH ratio value gave 58% probability to achieve SCC first. Thus, it could be concluded, that for a patient to have a probability of healing first, the metabolic rate of the standard dose INH should be sufficiently high, while non-efficient INH metabolic rate is not favourable. Also, quite surprisingly, our results from the time-to-event analysis showed that intermediate acetylators recovered more rapidly than slow acetylators; however, the results of univariate analysis did not reach statistically significant difference between NAT2 phenotypes and tSCC. Somewhere controversially, several studies have shown that low INH C_max_ and/or AUC values in rapid acetylators were associated with longer tSCC and a higher likelihood of failure or relapse; however, other studies, and also this study, found no association between the INH concentrations and treatment outcomes (Sekaggya-Wiltshire, von Braun, et al., 2018). INH plasma half-life is approximately 70 min in fast and 2–5 h in slow acetylators (Goodman et al., 2006), thus INH C_max_ measurements at a single 2 h time point may not fully reflect INH exposure for all patients. Thus, in our study, plasma concentrations of AcINH and INA were measured, and INH metabolic ratio values were used in addition to INH C_max_ and AUC values, which could provide more accurate assessments of the data.

On the other hand, inconsistency of the results may be due to the variability of the study population, the involvement of different factors, and the study design. TB treatment outcome should be considered as multifactorial event, considering multiple factors such as bacillary load, *M. tuberculosis* strain, individual measurement or local population estimate of the infecting strain minimum inhibitory concentration (MIC) values, treatment adherence, insufficient drug absorption, drug-drug interactions, drug concentration at the site of TB localization, age, gender, comorbid conditions, and immune and nutritional status of the patient (Colangeli et al., 2018; Perumal et al., 2020). Importantly, it was reported recently that INH concentrations were 14.6-fold higher in lung/airway epithelial lining fluid than blood plasma, thus accumulation of drugs at the site of disease may explain the success of the first-line regimen (McCallum et al., 2021).

Our study had limitations. First, the number of cohort participants was relatively small, and we were not able to include individuals with fast NAT2 phenotype. Second, the study was conducted in the Latvian population with European ancestry, and the obtained data were not extrapolated to other ethnicities. Also, we were not able to include all possible patient-, disease-, and treatment-related factors, and there is a possibility that the statistical power of the analysis to identify all genetic polymorphisms/SNPs was not sufficient.

## 5 Conclusion

In conclusion, the results of our study brought closer the research on anti-TB drug pharmacology and its application in clinical practice. Our findings suggest that *CYP2E1*, but not *GSTM1*, significantly affects the INH pharmacokinetics along with *NAT2*. Also, significant associations between INH pharmacokinetic parameters and both incidence of hepatotoxicity and month two sputum culture positivity were observed. AcINH plasma level could serve as a biomarker for INH-related hepatotoxicity. These findings could have further implications in promotion of the concept of precision medicine in TB management. The inclusion of INH metabolite screening in TB therapeutic drug monitoring could be beneficial in clinical studies for determination of optimal dosing strategies.

## Data Availability

The original contributions presented in the study are publicly available. This data can be found here: European Nucleotide Archive, accession number PRJEB69677.
