# Supplementary material for: Effect of NAT2, GSTM1 and CYP2E1 genetic polymorphisms on plasma concentration of isoniazid and its metabolites in patients with tuberculosis, and the assessment of exposure-response relationships
Source: Front Pharmacol. 2024 Mar 22;15:1332752. doi: 10.3389/fphar.2024.1332752 (PMC10995391; doi:10.3389/fphar.2024.1332752)
Supplement: Supplementary file 1 [file DataSheet1.docx]

Supplementary Material

*Tables and Figures*

Effect of NAT2, GSTM1 and CYP2E1 genetic polymorphisms on plasma concentration of isoniazid and its metabolites in patients with tuberculosis, and the assessment of exposure-response relationships

Viktorija Ulanova ^1, 2^, Agnija Kivrane ^1, 2^, Anda Viksna ^2, 3^, Leonora Pahirko ^4^, Lauma Freimane ^1^, Darja Sadovska ^1, 2^, Iveta Ozere ^2, 3^, Andra Cirule ^3^, Eduards Sevostjanovs ^5^, Solveiga Grinberga ^5^, Dace Bandere ^2^, Renate Ranka ^1,2*^.

^1^ Laboratory of molecular microbiology, Latvian Biomedical Research and Study Centre, Riga, Latvia

^2^ Pharmacogenetics laboratory, Department of Pharmaceutical Chemistry, Riga Stradins University, Riga, Latvia

^3^ Centre of Tuberculosis and Lung Diseases, Riga East University Hospital, Upeslejas, Latvia

^4^ Faculty of Physics, Mathematics and Optometry, University of Latvia, Riga, Latvia

^5^ Latvian Institute of Organic Synthesis, Riga, Latvia

**List of supplementary material**

[Supplementary Table 1. Patients characteristics and data. 3](#_Toc149774293)

[Supplementary Table 2. *CYP2E1* gene sequencing data quality for the study sample set (n = 34). 5](#_Toc149774294)

[Supplementary Table 3. *CYP2E1* single nucleotide variant (SNV) characterisation using reference SNP (rs) reports accumulated in NCBI dbSNP database (https://www.ncbi.nlm.nih.gov/snp/) and Varsome The Human Genomics Community platform (https://varsome.com/). 6](#_Toc149774295)

[Supplementary Table 4. Factor association with sputum microscopy results. 15](#_Toc149774296)

[Supplementary Figure 1. Histogram reflecting changes in ALAT, ASAT, total, and conjugated bilirubin ratio values during the therapy among patients with tuberculosis. 16](#_Toc149774297)

[Supplementary Figure 2. XY plot showing the correlations between isoniazid dose (mg/kg) and INH pharmacokinetics parameters. 17](#_Toc149774298)

[Supplementary Figure 3. XY plot showing the correlations between patient age (years) and INH pharmacokinetics parameters. 18](#_Toc149774299)

[Supplementary Figure 4. XY plots showing correlation analysis results for isoniazid and two metabolites in blood plasma of patients with tuberculosis. Patient samples were stratified based on the GSTM1 genotype. 19](#_Toc149774300)

# Supplementary Table 1. Patients’ characteristics and data.

| NGS ID | Sex | Age | BMI,  kg/m2 | Smoking status | Self- reported daily alcohol intake | ALAT  before therapy, U/L | ASAT  before therapy, U/L | Total bilirubin before therapy, µmol/L | Conjugated (direct) bilirubin before therapy,  µmol/L | ALAT  during therapy, U/L | ASAT  during therapy, U/L | Total bilirubin during therapy, µmol/L | Conjugated (direct) bilirubin during therapy,  µmol/L | Sputum microscopy result | tSCC | TB outcome | NAT2  genotype | NAT2  phenotype | GSTM1  genotype | INH  Cmax 2h, µg/mL |
| --- | --- | --- | --- | --- | --- | --- | --- | --- | --- | --- | --- | --- | --- | --- | --- | --- | --- | --- | --- | --- |
| TB102 | Male | 51 | 22.65 | yes | no | 14 | 17 | 8.4 | 3.6 | 13 | 16 | 2.5 | 1.3 | Positive | 77 | Cured | *5B/*5B | SA | plus | 2.12 |
| TB105 | Male | 19 | 17 | no | no | 14 | 18 | 8.2 | 4 | 20 | 18 | 3.6 | 2.5 | Negative | NA | Treatment completed | *5B/*6A | SA | plus | 2.27 |
| TB110 | Female | 23 | 17.31 | no | no | 16 | 12 | 12.2 | 5.5 | 12 | 17 | 6.5 | 3.4 | Negative | NA | Lost to follow-up | *5B/*5B | SA | plus | 3.64 |
| TB113 | Male | 57 | 21.13 | yes | yes | 6 | 11 | 6.9 | 3 | 12 | 14 | 2.5 | 1.5 | Positive | 197 | Cured | *5B/*5C | SA | plus | 4.26 |
| TB127 | Male | 46 | 18.87 | yes | yes | 13.4 | 18 | 5 | 3 | 18 | 22 | 5.7 | 2.7 | Positive | 112 | Cured | *5B/*6A | SA | plus | 0.74 |
| TB130 | Female | 43 | 23.03 | no | no | 14 | 15 | 7.4 | 3.4 | 443 | 146 | 7.6 | 3.4 | Negative | NA | Treatment completed | *6A/*6A | SA | plus | 4.75 |
| TB225 | Female | 49 | 18.8 | yes | no | 7 | 12 | 10.7 | 3.9 | 9 | 13 | 7 | 4.2 | Negative | 29 | Cured | *5B/*5A | SA | plus | 2.26 |
| TB237 | Male | 39 | 17.3 | yes | yes | 33 | 27 | 13.5 | 9 | 20 | 35 | 5.9 | 3.9 | Positive | 24 | Cured | *5B/*5B | SA | plus | 4.44 |
| TB204 | Male | 47 | 21.1 | yes | yes | 8 | 14 | 4.8 | 2.2 | 2560 | 1352 | 4 | 3.4 | Positive | 55 | Cured | *5C/*6A | SA | plus | 2.41 |
| TB232 | Male | 77 | 31.7 | no | no | 14 | 20 | 11 | 3.9 | 14 | 22 | 8.1 | 4 | Positive | 37 | Cured | *5B/*6A | SA | plus | 3.26 |
| TB106 | Male | 31 | 22.26 | yes | no | 18 | 22 | 5.2 | 2.6 | 17 | 20 | 2.5 | 1.6 | Positive | 26 | Cured | *5B/*5B | SA | null | 2.61 |
| TB108 | Male | 59 | 20.83 | yes | yes | 9 | 18 | 6.1 | 3.2 | 16 | 21 | 2.4 | 1.3 | Negative | 42 | Cured | *5B/*6A | SA | null | 0.11 |
| TB112 | Male | 63 | 16.04 | yes | yes | 62 | 134 | 14 | 8 | 172 | 239 | 21.3 | 29.4 | Positive | 83 | Cured | *5B/*5C | SA | null | 2.62 |
| TB115 | Male | 52 | 17.36 | yes | yes | 8 | 18 | 9 | 4.4 | 13 | 20 | 3.6 | 2.3 | Negative | NA | Treatment completed | *6A/*6A | SA | null | 3.91 |
| TB116 | Male | 32 | 22.41 | yes | no | 28 | 48 | 13.4 | 5.5 | 24 | 44 | 4.9 | 2.5 | Negative | NA | Lost to follow-up | *5B/*7B | SA | null | 3.95 |
| TB119 | Female | 56 | 25.21 | no | no | 49 | 26 | 10.9 | 3.8 | 207 | 221 | 4.6 | 2.1 | Negative | 65 | Cured | *6J/*7B | SA | null | 1.18 |
| TB124 | Male | 54 | 20.53 | yes | no | 6 | 3 | 4.2 | 1.9 | 9 | 25 | 2.5 | 1.5 | Positive | 85 | Cured | *6A/*6A | SA | null | 1.31 |
| TB125 | Male | 34 | 18.83 | yes | yes | 15 | 24 | 8 | 3 | 15 | 22 | 3.1 | 2.3 | Positive | 66 | Cured | *5B/*5B | SA | null | 3.94 |
| TB132 | Female | 63 | 25.95 | yes | no | 13 | 18 | 6 | 4.8 | 15 | 28 | 4.6 | 1.9 | Positive | 42 | Cured | *5B/*6A | SA | null | 2.47 |
| TB206 | Male | 49 | 19 | yes | no | 20 | 31 | 7.4 | 5.1 | 17 | 20 | 4.1 | 3.4 | Positive | 77 | Cured | *5B/*5A | SA | null | 5.12 |
| TB221 | Male | 57 | 22.8 | no | no | 11 | 34 | 5.3 | 1.6 | 12 | 33 | 5.8 | 5.1 | Positive | 96 | Cured | *5B/*5C | SA | null | 4.03 |
| TB231 | Male | 33 | 19.9 | yes | no | 17 | 9 | 5.4 | 2.1 | 11 | 12 | 8 | 4.4 | Positive | 57 | Cured | *6A/*6A | SA | null | 6.47 |
| TB235 | Male | 38 | 20.1 | yes | no | 43 | 71 | 3.6 | 2.4 | 44 | 40 | 4.5 | 3.3 | Negative | 64 | Cured | *5B/*5B | SA | null | 4.69 |
| TB118 | Male | 44 | 20.05 | yes | no | 27 | 42 | 8.2 | 4 | 16 | 22 | 7.9 | 2.9 | Negative | 49 | Cured | *4 /*7B | IA | plus | 1.88 |
| TB129 | Male | 32 | 24.73 | yes | yes | 21 | 18 | 7.8 | 9 | 19 | 26 | 9.5 | 5.9 | Negative | 41 | Cured | *4 /*5B | IA | plus | 2.78 |
| TB230 | Male | 44 | 22.3 | no | no | 16 | 18 | 10.9 | 4.3 | 16 | 16 | 5.3 | 2.8 | Negative | 46 | Cured | *4 /*6A | IA | plus | 2.29 |
| TB228 | Male | 38 | 24.5 | yes | no | 26 | 27 | 6.3 | 2.7 | 29 | 19 | 3.6 | 2.3 | Positive | 92 | Cured | *4 /*5B | IA | plus | 1.37 |
| TB104 | Male | 40 | 26.15 | yes | yes | 20 | 12 | 5.2 | 3 | 30 | 35 | 5.7 | 3 | Positive | 15 | Cured | *4 /*6A | IA | null | 1.65 |
| TB122 | Female | 55 | 21.56 | no | no | 23 | 21 | 7 | 2 | 30 | 23 | 2.5 | 2 | Negative | 27 | Cured | *4 /*5B | IA | null | 0.56 |
| TB126 | Male | 58 | 44.73 | no | no | 25 | 18 | 6 | 4 | 25 | 20 | 7.5 | 3.4 | Negative | NA | Cured | *4 /*6A | IA | null | 1.14 |
| TB131 | Male | 46 | 17.65 | yes | no | 18 | 15 | 5 | 2 | 17 | 20 | 5.1 | 2.2 | Negative | 46 | Cured | *4 /*5C | IA | null | 1.98 |
| TB133 | Male | 44 | 17.96 | no | no | 10 | 13 | 7.7 | 3.1 | 9 | 18 | 9.1 | 4.2 | Positive | 84 | Cured | *4 /*5B | IA | null | 2.70 |
| TB236 | Male | 46 | 18.6 | no | yes | 17 | 33 | 7.9 | 3.8 | 17 | 21 | 3.7 | 1 | Positive | 11 | Cured | *4 /*6A | IA | null | 2.23 |
| TB226 | Male | 54 | 18.4 | no | no | 30 | 27 | 5.1 | 3.1 | 14 | 16 | 2.2 | 1.5 | Positive | 21 | Cured | *12A/5A | IA | null | 1.90 |

Abbreviations: TB, tuberculosis; NGS, next generation sequencing; BMI, body mass index; ALAT, alanine aminotransferase; ASAT, aspartate aminotransferase; tSCC, time to sputum culture conversion; NAT2, N-acetyltransferase 2; SA, slow acetylator; IA, intermediate acetylator; GSTM1, glutathione S-transferase mu 1 class; INH, isoniazid; C_max_, maximum concentration; NA, not applicable.

# Supplementary Table 2. *CYP2E1* gene sequencing data quality for the study sample set (n = 34).

| Patient ID | Number of reads mapped | Average read length (bp) | Mean base quality score | Mean read depth | ≥Tenfold coverage† (%) | No coverage‡ (%) |
| --- | --- | --- | --- | --- | --- | --- |
| TB102 | 130961 | 162 | 37.6 | 455 | 98.6 | 0.32 |
| TB104 | 114780 | 161 | 37.7 | 622 | 99.5 | 0.01 |
| TB105 | 117132 | 164 | 37.6 | 558 | 99.4 | 0.0 |
| TB106 | 116019 | 159 | 37.6 | 576 | 98.8 | 0.02 |
| TB108 | 117943 | 162 | 37.5 | 540 | 99.0 | 0.16 |
| TB110 | 115422 | 163 | 37.5 | 498 | 98.9 | 0.0 |
| TB112 | 98905 | 164 | 37.5 | 469 | 98.7 | 0.0 |
| TB113 | 104597 | 160 | 37.5 | 467 | 99.1 | 0.07 |
| TB115 | 103008 | 160 | 37.5 | 530 | 99.3 | 0.0 |
| TB116 | 112917 | 161 | 37.5 | 537 | 99.0 | 0.04 |
| TB118 | 119264 | 158 | 37.5 | 566 | 98.6 | 0.11 |
| TB119 | 121566 | 159 | 37.6 | 468 | 99.1 | 0.0 |
| TB130 | 74931 | 162 | 37.8 | 263 | 99.3 | 0.05 |
| TB122 | 125017 | 161 | 37.6 | 647 | 99.2 | 0.08 |
| TB124 | 118351 | 159 | 37.6 | 435 | 98.6 | 0.55 |
| TB126 | 123154 | 167 | 37.6 | 579 | 98.8 | 0.02 |
| TB127 | 119206 | 163 | 37.6 | 495 | 99.3 | 0.03 |
| TB129 | 128848 | 161 | 37.6 | 636 | 99.0 | 0.01 |
| TB131 | 77674 | 159 | 37.8 | 321 | 98.6 | 0.25 |
| TB132 | 74579 | 158 | 37.8 | 267 | 99.21 | 0.06 |
| TB133 | 64983 | 166 | 37.8 | 286 | 98.5 | 0.01 |
| TB206 | 393477 | 159 | 37.4 | 222 | 98.4 | 0.01 |
| TB221 | 474282 | 155 | 37.2 | 227 | 98.4 | 0.52 |
| TB231 | 457025 | 159 | 37.4 | 227 | 98.3 | 0.21 |
| TB225 | 398577 | 157 | 37.3 | 248 | 98.5 | 0.42 |
| TB237 | 430795 | 163 | 37.5 | 230 | 98.3 | 0.25 |
| TB235 | 400647 | 167 | 37.5 | 203 | 98.8 | 0.22 |
| TB125 | 119442 | 162 | 37.6 | 546 | 99.5 | 0.0 |
| TB204 | 362081 | 163 | 37.4 | 207 | 97.8 | 0.73 |
| TB228 | 385171 | 166 | 37.3 | 226 | 98.6 | 0.0 |
| TB230 | 334309 | 153 | 37.3 | 221 | 98.9 | 0.08 |
| TB232 | 484175 | 165 | 37.4 | 219 | 98.0 | 0.23 |
| TB236 | 485948 | 155 | 37.4 | 273 | 98.9 | 0.55 |
| TB226 | 387123 | 163 | 37.4 | 222 | 98.3 | 0.23 |

†Percentage of the target fragment (11,761 bp) with at least tenfold coverage, genomic coordinates chr10:133524920-133539123

‡Percentage of the target fragment (11,761 bp) with zero coverage, genomic coordinates chr10: 133524920-133539123

# Supplementary Table 3. *CYP2E1* single nucleotide variant (SNV) characterisation using reference SNP (rs) reports accumulated in NCBI dbSNP database (https://www.ncbi.nlm.nih.gov/snp/) and Varsome The Human Genomics Community platform (https://varsome.com/).

| **SNP position** | **Position** | **SNP ID** | **Allelic variant** | **Number of patients** | **Functional classification** | **MAF** | ***p* HWE** | **Clinical significance (ACMG-referenced)** | **SNV was associated with drug metabolism and adverse drug reactions, reference** |
| --- | --- | --- | --- | --- | --- | --- | --- | --- | --- |
| Exonic variants | chr10:133532171 | rs6413419  (G>A) | *CYP2E1*4* | 3 | Missense | A(0.04) | 0.999 | Benign (BA1, BP1, BP4, BP6 rules) | García-Suástegui et al., 2017 |
|  |  |  |  |  |  | G(0.96) |  |  |  |
|  | chr10:133537858 | rs2515641  (C>T) |  | 9 | Missense | T(0.13) | 0.785 | Uncertain significance (BP1, PM2 rules) | Richardson et al., 2018 |
|  |  |  |  |  |  | C(0.87) |  |  |  |
|  | chr10:133527459 | rs900694954*  (A>G) |  | 1 | Missense | G(0.01) | 1.000 | Likely benign (BP1, BP4, PM2) |  |
|  |  |  |  |  |  | A(0.99) |  |  |  |
| Intronic variants | chr10:133528756 | rs943975  (C>T) |  | 34 | Non-coding | T(0.88) | 1.000 | Benign (BA1, BP4 rules) |  |
|  |  |  |  |  |  | C(0.12) |  |  |  |
|  | chr10:133528811 | rs1536828  (G>C) |  | 34 | Non-coding | C(0.79) | 0.800 | Benign (BA1, BP4 rules) |  |
|  |  |  |  |  |  | G(0.21) |  |  |  |
|  | chr10:133530234 | rs915906  (C>T) |  | 34 | Non-coding | T(0.84) | 0.791 | Benign (BA1, BP4 rules) |  |
|  |  |  |  |  |  | C(0.16) |  |  |  |
|  | chr10:133531025 | rs2771202  (T>C) |  | 34 | Non-coding | C(1.00) | 1.000 | Likely benign (BP4, PM2 rules) |  |
|  |  |  |  |  |  | T(0.00) |  |  |  |
|  | chr10:133534029 | rs943976  (A>G) |  | 34 | Non-coding | G(1.00) | 1.000 | Benign (BA1, BP4 rules) |  |
|  |  |  |  |  |  | A(0.00) |  |  |  |
|  | chr10:133534683 | rs8192776  (T>C) |  | 28 | Non-coding | C(0.72) | 0.177 | Benign (BA1, BP4 rules) |  |
|  |  |  |  |  |  | T(0.28) |  |  |  |
|  | chr10:133535253 | rs2864985  (C>G) |  | 32 | Non-coding | G(0.87) | 1.000 | Benign (BA1, BP4 rules) |  |
|  |  |  |  |  |  | C(0.13) |  |  |  |
|  | chr10:133536033 | rs1410897  (G>A) |  | 34 | Non-coding | A(0.88) | 1.000 | Benign (BA1, BP4 rules) |  |
|  |  |  |  |  |  | G(0.12) |  |  |  |
|  | chr10:133536297 | rs1329149  (T>C) |  | 34 | Non-coding | C(0.88) | 0.776 | Likely benign (BP4, PM2 rules) |  |
|  |  |  |  |  |  | T(0.12) |  |  |  |
|  | chr10:133536410 | rs12761234  (C>T) |  | 34 | Non-coding | T(0.87) | 0.785 | Likely benign (BP4 rules) |  |
|  |  |  |  |  |  | C(0.13) |  |  |  |
|  | chr10:133537367 | rs8192777  (G>T) |  | 34 | Non-coding | T(0.87) | 0.785 | Likely benign (BP4, PM2 rules) |  |
|  |  |  |  |  |  | G(0.13) |  |  |  |
|  | chr10:133537633 | rs2070676  (G>C) | *CY2E1*1B* | 34 | Non-coding | C(0.87) | 0.785 | Likely benign (BP4, PM2 rules) | Yu et al., 2019 |
|  |  |  |  |  |  | G(0.13) |  |  |  |
|  | chr10:133537670 | rs2070677  (T>A) |  | 34 | Non-coding | A(0.87) | 0.785 | Benign (BA1, BP4 rules) |  |
|  |  |  |  |  |  | T(0.13) |  |  |  |
|  | chr10:133538509 | rs2515642  (C>T) |  | 34 | Non-coding | T(0.81) | 0.609 | Likely benign (BP4, PM2 rules) |  |
|  |  |  |  |  |  | C(0.19) |  |  |  |
|  | chr10:133538572 | rs2480259  (C>T) |  | 34 | Non-coding | T(0.81) | 0.609 | Benign (BA1, BP4 rules) |  |
|  |  |  |  |  |  | C(0.19) |  |  |  |
|  | chr10:133538596 | rs2480258  (C>T) |  | 34 | Non-coding | T(0.81) | 0.609 | Benign (BA1, BP4 rules) |  |
|  |  |  |  |  |  | C(0.19) |  |  |  |
|  | chr10:133538649 | rs2249694  (A>G) |  | 34 | Non-coding | G(0.79) | 0.615 | Likely benign (BP4, PM2 rules) |  |
|  |  |  |  |  |  | A(0.21) |  |  |  |
|  | chr10:133538664 | rs2249695  (T>C) |  | 34 | Non-coding | C(0.82) | 0.609 | Benign (BA1, BP4 rules) |  |
|  |  |  |  |  |  | T(0.18) |  |  |  |
|  | chr10:133535722 | rs743534  (C>A) |  | 34 | Non-coding | A(0.87) | 0.785 | Benign (BA1, BP4 rules) |  |
|  |  |  |  |  |  | C(0.13) |  |  |  |
|  | chr10:133533455 | rs9159089  (G>A) |  | 12 | Non-coding | A(0.18) | 0.796 | Benign (BA1, BP4 rules) | Richardson et al., 2018 |
|  |  |  |  |  |  | G(0.82) |  |  |  |
|  | chr10:133529079 | rs8192769  (C>T) |  | 4 | Non-coding | T(0.06) | 1.000 | Benign (BA1, BP4 rules) |  |
|  |  |  |  |  |  | C(0.94) |  |  |  |
|  | chr10:133529140 | rs8192770  (G>A) |  | 6 | Non-coding | A(0.09) | 1.000 | Benign (BA1, BP4 rules) |  |
|  |  |  |  |  |  | G(0.91) |  |  |  |
|  | chr10:133531207 | rs8192772  (T>C) |  | 4 | Non-coding | C(0.06) | 1.000 | Benign (BA1, BP4 rules) |  |
|  |  |  |  |  |  | T(0.94) |  |  |  |
|  | chr10:133533192 | rs2070675  (C>T) |  | 10 | Non-coding | T(0.15) | 1.000 | Benign (BA1, BP4 rules) |  |
|  |  |  |  |  |  | C(0.85) |  |  |  |
|  | chr10:133533423 | rs915907  (C>A) |  | 7 | Non-coding | A(0.10) | 1.000 | Benign (BA1, BP4 rules) |  |
|  |  |  |  |  |  | C(0.90) |  |  |  |
|  | chr10:133534522 | rs8192775  (G>A) |  | 4 | Non-coding | A(0.07) | 0.855 | Benign (BA1, BP4 rules) | Richardson et al., 2018 |
|  |  |  |  |  |  | G(0.93) |  |  |  |
|  | chr10:133534524 | rs188765034  (A>T) |  | 2 | Non-coding | T(0.04) | 0.614 | Benign (BS1, BS2, BP4 rules) |  |
|  |  |  |  |  |  | A(0.96) |  |  |  |
|  | chr10:133534753 | rs7092584  (C>T) |  | 4 | Non-coding | T(0.06) | 1.000 | Benign (BA1, BP4 rules) |  |
|  |  |  |  |  |  | C(0.94) |  |  |  |
|  | chr10:133535040 | rs6413432  (T>A) | *CYP2E1*6* | 4 | Non-coding | A(0.06) | 1.000 | Benign (BA1, BP4, BP6 rules) | Wu et al., 2019 |
|  |  |  |  |  |  | T(0.94) |  |  |  |
|  | chr10:133535075 | rs2864987  (A>G) |  | 4 | Non-coding | G(0.06) | 1.000 | Benign (BA1, BP4 rules) |  |
|  |  |  |  |  |  | A(0.94) |  |  |  |
|  | chr10:133535169 | rs2864986  (C>A) |  | 4 | Non-coding | A(0.09) | 0.155 | Benign (BA1, BP4 rules) |  |
|  |  |  |  |  |  | C(0.91) |  |  |  |
|  | chr10:133535282 | rs2011661  (C>T) |  | 4 | Non-coding | T(0.07) | 0.855 | Benign (BA1, BP4 rules) |  |
|  |  |  |  |  |  | C(0.93) |  |  |  |
|  | chr10:133535453 | rs2864984  (C>T) |  | 4 | Non-coding | T(0.06) | 1.000 | Benign (BA1, BP4 rules) |  |
|  |  |  |  |  |  | C(0.94) |  |  |  |
|  | chr10:133535863 | rs743535  (G>A) |  | 4 | Non-coding | A(0.06) | 1.000 | Benign (BA1, BP4 rules) |  |
|  |  |  |  |  |  | G(0.94) |  |  |  |
|  | chr10:133534371 | rs375648847  (T>C) |  | 4 | Non-coding | C(0.07) | 0.855 | Likely benign (BP4, PM2 rules) |  |
|  |  |  |  |  |  | T(0.93) |  |  |  |
|  | chr10:133531075 | rs2854144  (A>G) |  | 1 | Non-coding | G(0.01) | 1.000 | Benign (BA1, BP4 rules) |  |
|  |  |  |  |  |  | A(0.99) |  |  |  |
|  | chr10:133532307 | rs6413421  (T>C) |  | 2 | Non-coding | C(0.03) | 1.000 | Benign (BA1, BP4, BP6 rules) |  |
|  |  |  |  |  |  | T(0.97) |  |  |  |
|  | chr10:133533201 | rs41299428  (T>C) |  | 2 | Non-coding | C(0.03) | 1.000 | Benign (BA1, BP4 rules) |  |
|  |  |  |  |  |  | T(0.97) |  |  |  |
|  | chr10:133528459 | rs72862138  (G>C) |  | 5 | Non-coding | C(0.07) | 1.000 | Likely benign (BP4, PM2 rules) |  |
|  |  |  |  |  |  | G(0.93) |  |  |  |
|  | chr10:133529278 | rs41299414  (C>G) |  | 5 | Non-coding | G(0.07) | 1.000 | Benign (BA1, BP4 rules) |  |
|  |  |  |  |  |  | C(0.93) |  |  |  |
|  | chr10:133537482 | rs28371747  (C>T) |  | 5 | Non-coding | T(0.07) | 1.000 | Benign (BA1, BP4, BP6 rules) |  |
|  |  |  |  |  |  | C(0.93) |  |  |  |
|  | chr10:133537724 | rs28371748  (C>G) |  | 1 | Non-coding | G(0.01) | 1.000 | Benign (BS1, BS2, BP4, BP6 rules) |  |
|  |  |  |  |  |  | C(0.99) |  |  |  |
|  | chr10:133537383 | rs1851418994*  (A>G) |  | 1 | Non-coding | G(0.01) | 1.000 | − |  |
|  |  |  |  |  |  | A(0.99) |  |  |  |
|  | chr10:133528431 | rs41299410  (C>T) |  | 1 | Non-coding | T(0.01) | 1.000 | Benign (BA1, BP4, BP6 rules) |  |
|  |  |  |  |  |  | C(0.99) |  |  |  |
|  | chr10:133530679 | rs41299420  (C>T) |  | 3 | Non-coding | T(0.04) | 1.000 | Likely benign (BP4, PM2 rules) |  |
|  |  |  |  |  |  | C(0.96) |  |  |  |
|  | chr10:133530877 | rs41299422  (G>A) |  | 4 | Non-coding | A(0.06) | 1.000 | Benign (BA1, BP4 rules) |  |
|  |  |  |  |  |  | G(0.94) |  |  |  |
|  | chr10:133531187 | rs41258504  (C>T) |  | 4 | Non-coding | T(0.06) | 1.000 | Benign (BA1, BP4 rules) |  |
|  |  |  |  |  |  | C(0.94) |  |  |  |
|  | chr10:133531319 | rs7088600*  (T>A) |  | 4 | Non-coding | A(0.06) | 1.000 | Benign (BA1, BP4 rules) |  |
|  |  |  |  |  |  | T(0.94) |  |  |  |
|  | chr10:133529731 | rs191586700  (G>A) |  | 2 | Non-coding | A(0.03) | 1.000 | Benign (BS1, BS2, BP4 rules) |  |
|  |  |  |  |  |  | G(0.97) |  |  |  |
|  | chr10:133530502 | rs9919379  (G>A) |  | 2 | Non-coding | A(0.03) | 1.000 | Benign (BA1, BP4 rules) |  |
|  |  |  |  |  |  | G(0.97) |  |  |  |
|  | chr10:133531889 | rs9919378  (G>T) |  | 3 | Non-coding | T(0.04) | 1.000 | Benign (BA1, BP4, BP6 rules) |  |
|  |  |  |  |  |  | G(0.96) |  |  |  |
|  | chr10:133532099 | rs9919386  (G>A) |  | 3 | Non-coding | A(0.04) | 1.000 | Benign (BA1, BP4, BP6 rules) |  |
|  |  |  |  |  |  | G(0.96) |  |  |  |
|  | chr10:133532494 | rs12254222  (C>T) |  | 3 | Non-coding | T(0.04) | 1.000 | Likely benign (BP4, PM2 rules) |  |
|  |  |  |  |  |  | C(0.96) |  |  |  |
|  | chr10:133528086 | rs1490922141*  (C>T) |  | 1 | Non-coding | T(0.01) | 1.000 | Likely benign (BS1, BP4 rules) |  |
|  |  |  |  |  |  | C(0.99) |  |  |  |
|  | chr10:133532307 | rs767704036*  (C>G) |  | 2 | Non-coding | G(0.03) | 1.000 | Likely benign (BP4, PM2 rules) |  |
|  |  |  |  |  |  | C(0.97) |  |  |  |
|  | chr10:133538603 | rs1851435078*  (A>G) |  | 1 | Non-coding | G(0.01) | 1.000 | − |  |
|  |  |  |  |  |  | A(0.99) |  |  |  |
|  | chr10:133532426 | rs568483402  (A>G) |  | 1 | Non-coding | G(0.01) | 1.000 | Likely benign (BS2, BP4 rules) |  |
|  |  |  |  |  |  | A(0.99) |  |  |  |
|  | chr10:133527761 | rs78660337  (C>T) |  | 1 | Non-coding | T(0.01) | 1.000 | Likely benign (BS2, BP4 rules) |  |
|  |  |  |  |  |  | C(0.99) |  |  |  |
|  | chr10:133531392 | rs41283307  (C>T) |  | 1 | Non-coding | T(0.01) | 1.000 | Benign (BS1, BS2, BP4 rules) |  |
|  |  |  |  |  |  | C(0.99) |  |  |  |
|  | chr10:133536879 | rs900651577*  (G>A) |  | 1 | Non-coding | A(0.01) | 1.000 | Likely benign (BP4 rules) |  |
|  |  |  |  |  |  | G(0.99) |  |  |  |
| Multiple Nucleotide variation | chr10:133532513-133532514 | rs386749499*  (CA>TG) |  | 3 | Non-coding | TG(0.04) | 1.000 | Likely benign (BP4, PM2 rules) |  |
|  |  |  |  |  |  | CA(0.96) |  |  |  |
| 3`UTR variants | chr10:133539005 | rs2480257  (T>A) |  | 34 | Non-coding | A(0.66) | 0.039 | Benign (BA1, BP4 rules) | Zhu et al., 2018 |
|  |  |  |  |  |  | T(0.34) |  |  |  |
|  | chr10:133539010 | rs2480256  (A>G) |  | 34 | Non-coding | G(0.66) | 0.039 | Likely benign (BP4, PM2 rules) | Zhu et al., 2018 |
|  |  |  |  |  |  | A(0.34) |  |  |  |
|  | chr10:133538986 | rs7081484  (C>T) |  | 5 | Non-coding | T(0.09) | 1.000 | Likely benign (BP4, PM2 rules) |  |
|  |  |  |  |  |  | C(0.91) |  |  |  |
| Indels | chr10:133527363 | rs11445593  (C>CC) |  | 34 | Non-coding | CC(1.00) | 1.000 | Benign (BA1, BP4 rules) |  |
|  |  |  |  |  |  | C(0.00) |  |  |  |
|  | chr10:133534371 | rs1554902928*  (T>insCGTC) |  | 33 | Non-coding | insCGTC (0.97) | 0.366 | Uncertain significance (BP4. PM2) |  |
|  |  |  |  |  |  | T(0.03) |  |  |  |
|  | chr10:133536987 | rs76496008  (GATGGGTGGATGATGGGTGGATG> GATGGGTGGATGATGGGTGGATGATGGGTGGATG) |  | 33 | Non-coding | GATGGGTGGATGATGGGTGGATGATGGGTGGATG (0.97) | 0.366 | Benign (BA1, BP4 rules) |  |
|  |  |  |  |  |  | GATGGGTGGATGATGGGTGGATG (0.03) |  |  |  |
|  | chr10:133534869 | rs71016459  (TTTTTTTTTTT> TTTTTTTTT) |  | 34 | Non-coding | TTTTTTTTT  (1.00) | 1.000 | Benign (BA1, BP4 rules) |  |
|  |  |  |  |  |  | TTTTTTTTTTT  (0.00) |  |  |  |
|  | chr10:133538103 | rs10552252  (TGTTGTTGT  > TGTTGT) |  | 34 | Non-coding | TGTTGT (1.00) | 1.000 | Benign (BA1, BP4 rules) |  |
|  |  |  |  |  |  | TGTTGTTGT  (0.00) |  |  |  |
| Intergenic | chr10:133525618 | rs41299398  (C>T) |  | 9 | Non-coding | T(0.15) | 1.000 | Benign (BA1, BP4 rules) |  |
|  |  |  |  |  |  | C(0.85) |  |  |  |
|  | chr10:133525978 | rs117944666  (C>A) |  | 9 | Non-coding, canonical | A(0.14) | 1.000 | Benign (BA1, BP4 rules) |  |
|  |  |  |  |  |  | C(0.86) |  |  |  |
|  | chr10:133525830 | rs3813866  (A>T) |  | 3 | Non-coding, canonical | A(0.04) | 1.000 | Benign (BA1, BP4 rules) | Huang et al., 2012 |
|  |  |  |  |  |  | T(0.96) |  |  |  |
|  | chr10:133525881 | rs8192766  (T>G) |  | 4 | Non-coding, canonical | G(0.06) | 1.000 | Benign (BA1, BP4 rules) | Huang et al., 2012 |
|  |  |  |  |  |  | T(0.94) |  |  |  |
|  | chr10:133526004 | rs6413423  (T>G) |  | 3 | Non-coding, canonical | G(0.04) | 1.000 | Benign (BA1, BP4 rules) |  |
|  |  |  |  |  |  | T(0.96) |  |  |  |
|  | chr10:133525526 | rs560259488  (G>C) |  | 1 | Non-coding | C(0.01) | 1.000 | Likely benign (BP4, PM2 rules) |  |
|  |  |  |  |  |  | G(0.99) |  |  |  |
|  | chr10:133525740 | rs3813865  (G>C) |  | 1 | Non-coding | C(0.01) | 1.000 | Benign (BA1, BP4 rules) |  |
|  |  |  |  |  |  | G(0.99) |  |  |  |
|  | chr10:133525165 | rs138420667  (G>T) |  | 2 | Non-coding | T(0.03) | 1.000 | Benign (BS1, BS2, BP4 rules) |  |
|  |  |  |  |  |  | G(0.97) |  |  |  |
| Upstream | chr10:133527063 | rs2070673  (A>T) | *CYP2E1*7A* | 34 | Non-coding | T(0.85) | 0.695 | Benign (BA1, BP4 rules) | Richardson et al., 2018 |
|  |  |  |  |  |  | A(0.15) |  |  |  |
|  | chr10:133527325 | rs6413420  (G>T) |  | 2 | Non-coding | T(0.03) | 1.000 | Benign (BA1, BP4, BP6 rules) |  |
|  |  |  |  |  |  | G(0.97) |  |  |  |
|  | chr10:133526466 | rs3813870  (A>G) |  | 1 | Non-coding, canonical | G(0.01) | 1.000 | Benign (BA1, BP4 rules) |  |
|  |  |  |  |  |  | A(0.99) |  |  |  |
|  | chr10:133526589 | rs2031922  (T>C) |  | 3 | Non-coding, canonical | C(0.04) | 1.000 | Benign (BA1, BP4 rules) | Richardson et al., 2018 |
|  |  |  |  |  |  | T(0.96) |  |  |  |
|  | chr10:133527044 | rs2070672  (A>G) |  | 1 | Non-coding | G(0.01) | 1.000 | Benign (BA1, BP4, BP6 rules) |  |
|  |  |  |  |  |  | A(0.99) |  |  |  |

SNP ID was referenced by the NCBI dbSNP database (<https://www.ncbi.nlm.nih.gov/snp/>); Reference genome: GRCh38.p14, GCF 000001405.40;

Transcript used to locate region containing SNP: NM_000773.4

Abbreviatons: SNP, single nucleotide polymorphism; N/A, not applicable; HWE, Hardy-Weinberg equilibrium; MAF, minor allele frequency; UTR, untranslated region; ACMG, The American College of Medical Genetics and Genomics.

ACMG implemented rules:

BA1 – Allele frequency is >5% in the Exome Sequencing Project, 1000 Genomes Project, or Exome Aggregation Consortium (Benign, Stand Alone);

BP1 – Missense variant in a gene for which primarily truncating variants are known to cause disease (Benign, Supporting);

BP4 – Multiple lines of computational evidence suggest no impact on gene or gene product (conservation, evolutionary, splicing impact, etc.) (Benign, Supporting);

BP6 – Reputable source recently reports variant as benign, but the evidence is not available to the laboratory to perform an independent evaluation. (Benign, Supporting);

BS1 – Allele frequency is greater than expected for disorder (Benign, Strong);

BS2 – Observed in a healthy adult individual for a recessive (homozygous), dominant (heterozygous), or X-linked (hemizygous) disorder, with full penetrance expected at an early age (Benign, Strong);

PM2 = Absent from controls (or at extremely low frequency if recessive) in the Exome Sequencing Project, 1000 Genomes Project, or Exome Aggregation Consortium (Pathogenic, Moderate).

* SNP that was not applicable for further Linkage disequilibrium analysis due to absence in 1000 Genomes Project database for SNP pairwise comparisons.

**References of Supplementary Table3 listed in alphabetical order.**

García-Suástegui, W. A., Ramos-Chávez, L. A., Rubio-Osornio, M., Calvillo-Velasco, M., Atzin-Méndez, J. A., Guevara, J., & Silva-Adaya, D. (2017). The Role of CYP2E1 in the Drug Metabolism or Bioactivation in the Brain. *Oxidative medicine and cellular longevity*, *2017*. https://doi.org/10.1155/2017/4680732

Huang, X., Chen, L., Song, W., Chen, L., Niu, J., Han, X., Feng, G., He, L., & Qin, S. (2012). Systematic functional characterization of cytochrome P450 2E1 promoter variants in the Chinese Han population. *PloS one*, *7*(7), e40883. https://doi.org/10.1371/journal.pone.0040883

Richardson, M., Kirkham, J., Dwan, K., Sloan, D. J., Davies, G., & Jorgensen, A. L. (2018). CYP genetic variants and toxicity related to anti-tubercular agents: a systematic review and meta-analysis. *Systematic reviews*, *7*(1), 1-15. <https://doi.org/10.1186/s13643-018-0861-z>

Wu, Z., Liu, Q., Wang, L., Zheng, M., Guan, M., Zhang, M., Zhao, W., Wang, C., Lu, S., Cheng, J., & Leng, S. (2019). The essential role of CYP2E1 in metabolism and hepatotoxicity of N, N-dimethylformamide using a novel C yp2e1 knockout mouse model and a population study. *Archives of Toxicology*, *93*, 3169-3181. <https://doi.org/10.1007/s00204-019-02567-7>

Yu, Y.-Y., Tsao, S.-M., Yang, W.-T., Huang, W.-C., Lin, C.-H., Chen, W.-W., Yang, S.-F., Chiou, H.-L., & Huang, Y.-W. (2020). Association of drug metabolic enzyme genetic polymorphisms and adverse drug reactions in patients receiving rifapentine and isoniazid therapy for latent tuberculosis. *International Journal of Environmental Research and Public Health*, *17*(1), 210. <https://doi.org/10.3390/ijerph17010210>

Zhu, L., He, Y., Niu, F., Yan, M., Li, J., Yuan, D., & Jin, T. (2018). Polymorphisms of drug-metabolizing enzyme CYP2E1 in Chinese Uygur population. *Medicine*, *97*(7). https://doi.org/10.1097/MD.0000000000009970

# Supplementary Table 4. Factor association with sputum microscopy results.

|  |  | Sputum microscopy result, N/Total (%) | |
| --- | --- | --- | --- |
|  |  | Positive | Negative |
| Biological sex | Male | 18/19 (94.7) | 10/15 (66.7) |
|  | Female | 1/19 (5.3) | 5/15 (33.3) |
|  | *p*-value | 0.0663 |  |
| Smoking status | Non-smoker | 2/19 (10.5) | 7/15 (46.7) |
|  | Smoker | 17/19 (89.5) | 8/15 (53.3) |
|  | *p*-value | **0.0252** |  |
| BMI | Underweigt | 4/19 (21.1) | 4/15 (26.7) |
|  | Normal | 12/19 (63.2) | 9/15 (60.0) |
|  | Overweight | 3/19 (15.8) | 2/15 (13.3) |
|  | *p*-value | 0.923 |  |
| Daily alcohol intake | Yes | 8/19 (42.1) | 3/15 (20.0) |
|  | No | 11/19 (57.9) | 12/15 (80.0) |
|  | *p*-value | 0.2714 |  |
| Mean age (±SD) |  | 48.58 (±11.81) | 43.33 (±12.43) |
|  | *p*-value | 0.2179 |  |

Abbreviations: BMI, body mass index; SD, standard deviation.


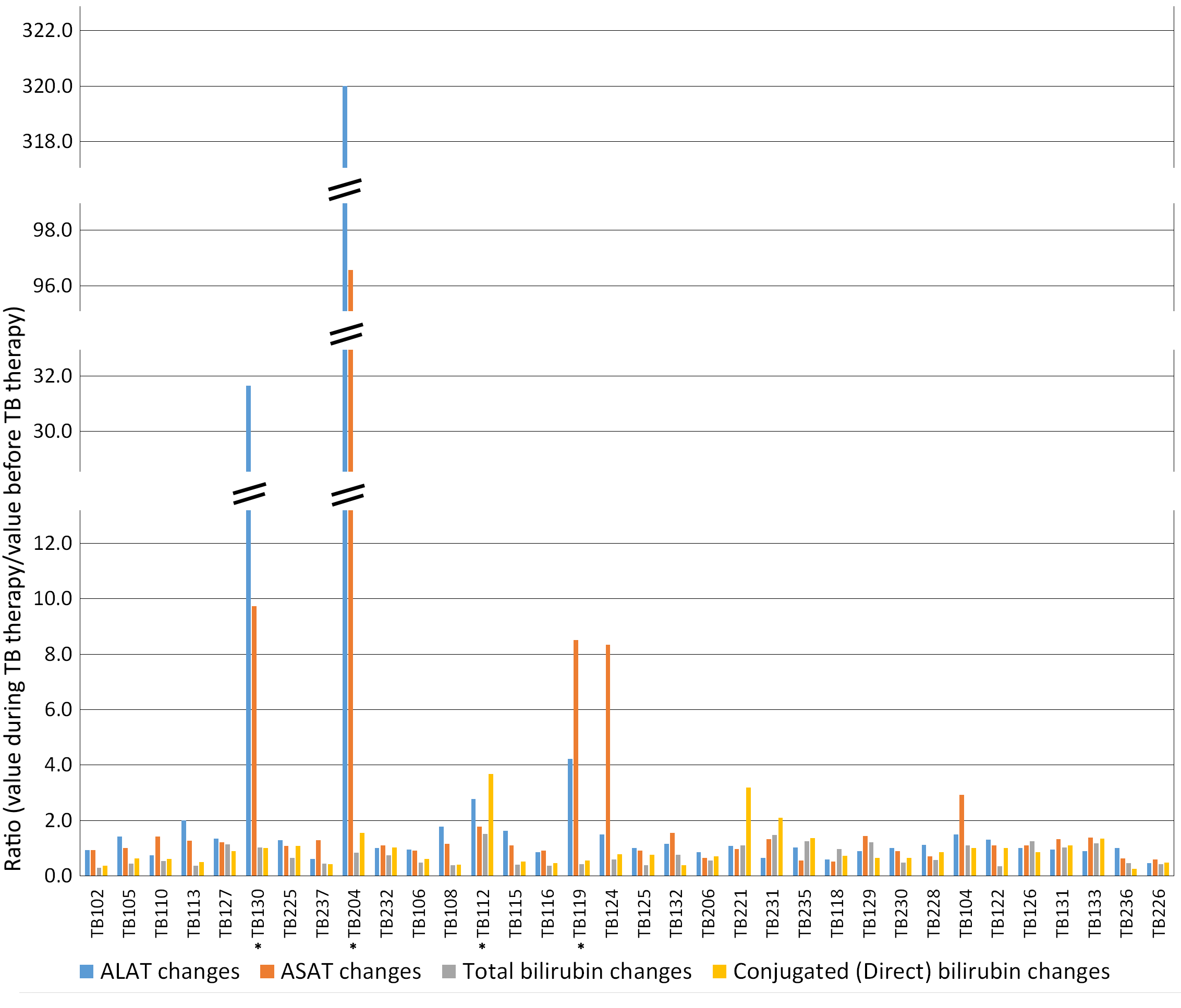


# Supplementary Figure 1. Histogram reflecting changes in ALAT, ASAT, total, and conjugated bilirubin ratio values during the therapy among patients with tuberculosis.

The colour scales for each liver biochemistry test parameter studied are arranged along the horizontal axis, and the liver biochemistry test ratios are arranged along the vertical axis. Each bar of the colour scale corresponds to one subject. Dots represent the patients who experienced hepatotoxicity.

Abbreviations: TB, tuberculosis; ALAT, alanine aminotransferase; ASAT, aspartate aminotransferase.


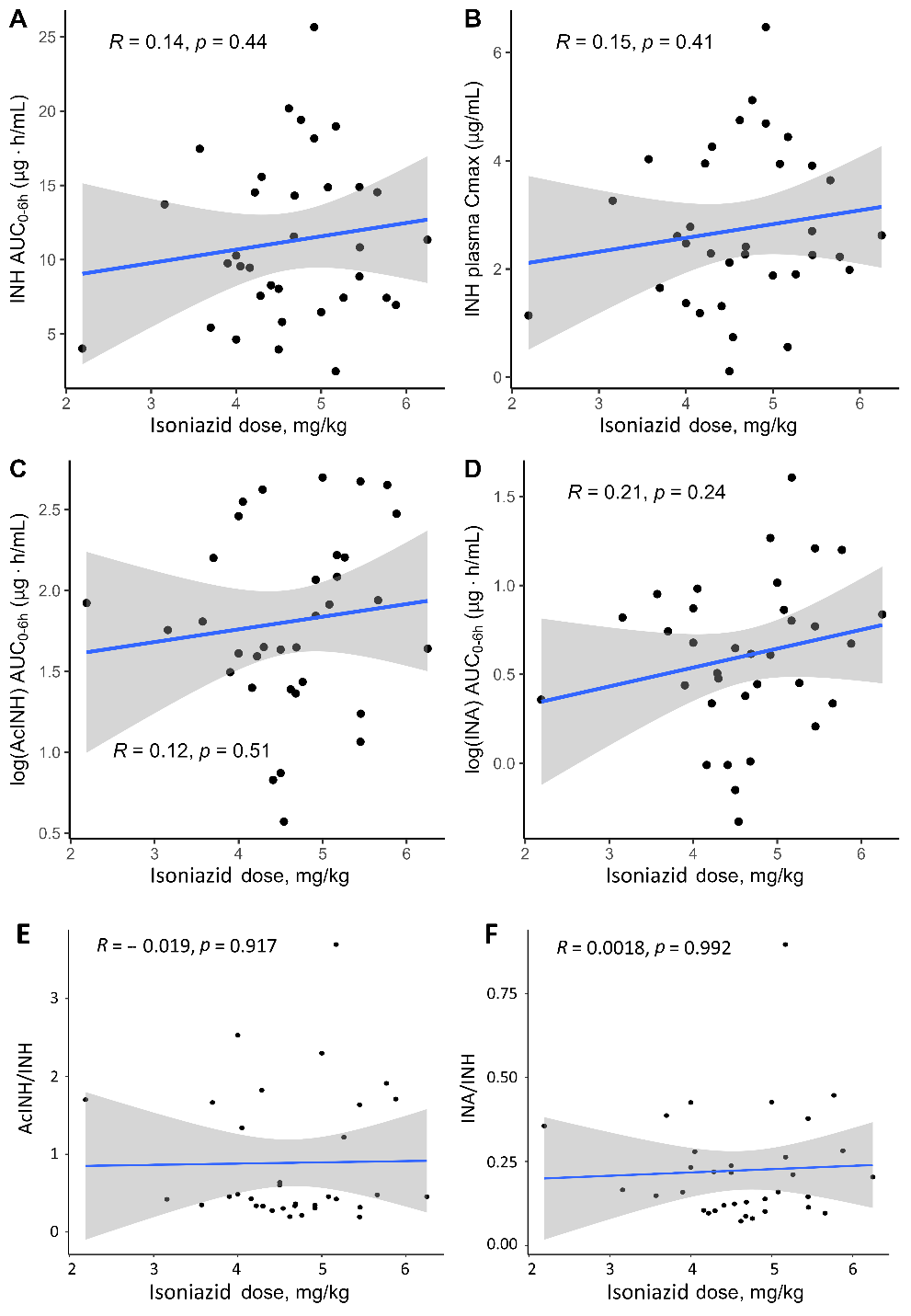


# Supplementary Figure 2. XY plot showing the correlations between isoniazid dose (mg/kg) and INH pharmacokinetics parameters.

(A) Dose versus INH AUC_0-6h_ (µg·h /mL). (B) Dose versus INH Cmax (2 h, µg/mL). (C) Dose versus AcINH AUC_0-6h_ (µg·h /mL). (D) Dose versus INA AUC_0-6h_ (µg·h /mL). (E) Dose vesus AcINH/INH ratio. (F) Dose versus INA/INH ratio.

The solid lines represent the Pearson correlation coefficient. AcINH and INA AUC_0-6h_ values were log-trans formed before running the tests. Spearman correlation was used for non-normally distributed variables (AcINH/INH and INA/INH ratios). Shaded areas represent the 95% confidence interval of the observed concentration mean values. Dots represent the observed data for a single patient.

Abbreviations: TB, tuberculosis; INH, isoniazid; AcINH, acetylisoniazid; INA, izonicotinic acid; AUC, area under the concentration-time curve; C_max_, maximum concentration.


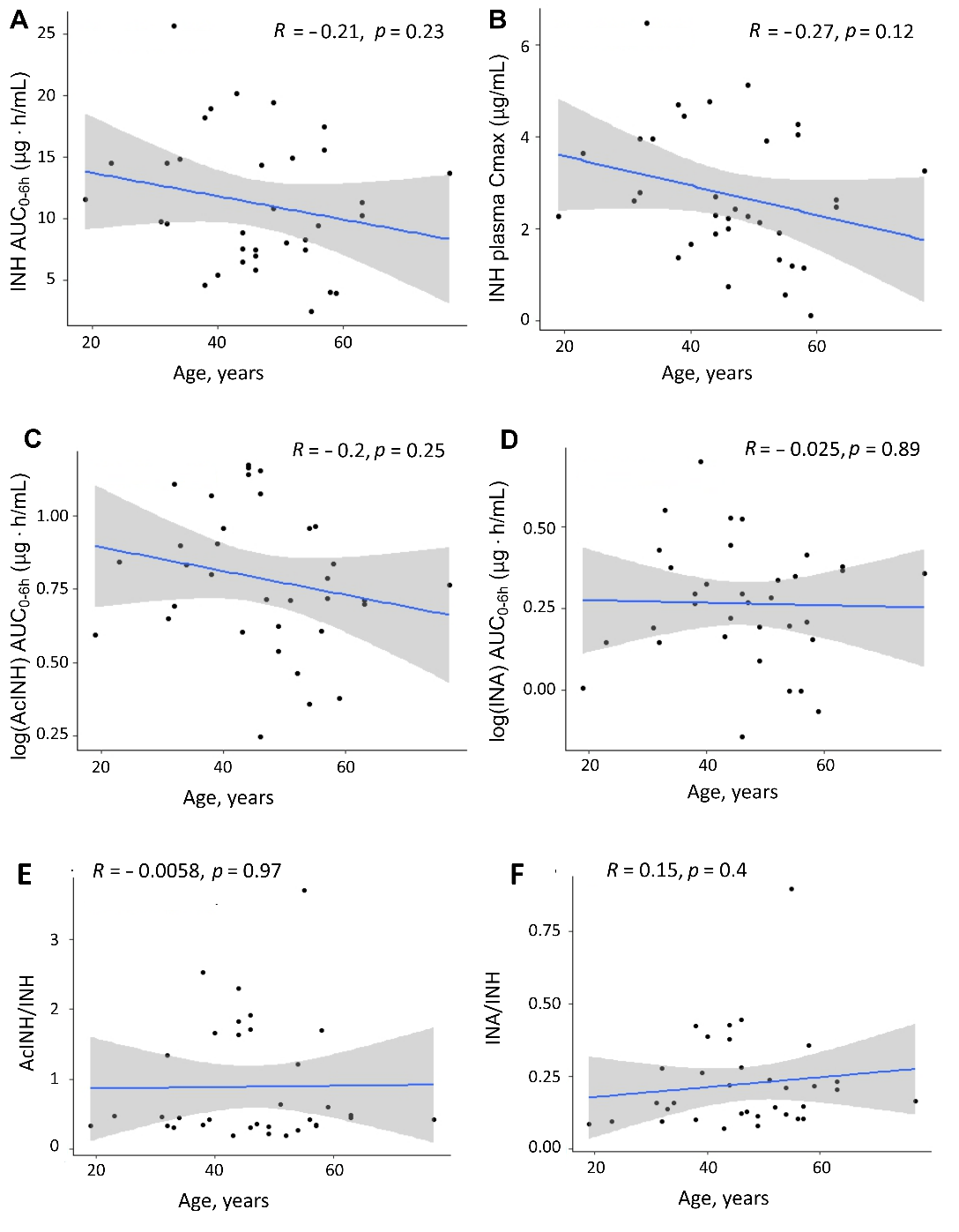


# Supplementary Figure 3. XY plot showing the correlations between patient age (years) and INH pharmacokinetics parameters.

(A) Age versus INH AUC_0-6h_ (µg·h /mL). (B) Age versus INH Cmax (2 h, µg/mL). (C) Age versus AcINH AUC_0-6h_ (µg·h /mL). (D) Age versus INA AUC_0-6h_ (µg·h /mL). (E) Age vesus AcINH/INH ratio. (F) Age versus INA/INH ratio.

The solid lines represent the Pearson correlation coefficient. AcINH and INA AUC_0-6h_ values were log-trans formed before running the tests. Spearman correlation was used for non-normally distributed variables (AcINH/INH and INA/INH ratios). Shaded areas represent the 95% confidence interval of the observed concentration mean values. Dots represent the observed data for a single patient.

Abbreviations: INH, isoniazid; AcINH, acetylisoniazid; INA, izonicotinic acid; AUC, area under the concentration-time curve; C_max_, maximum concentration.


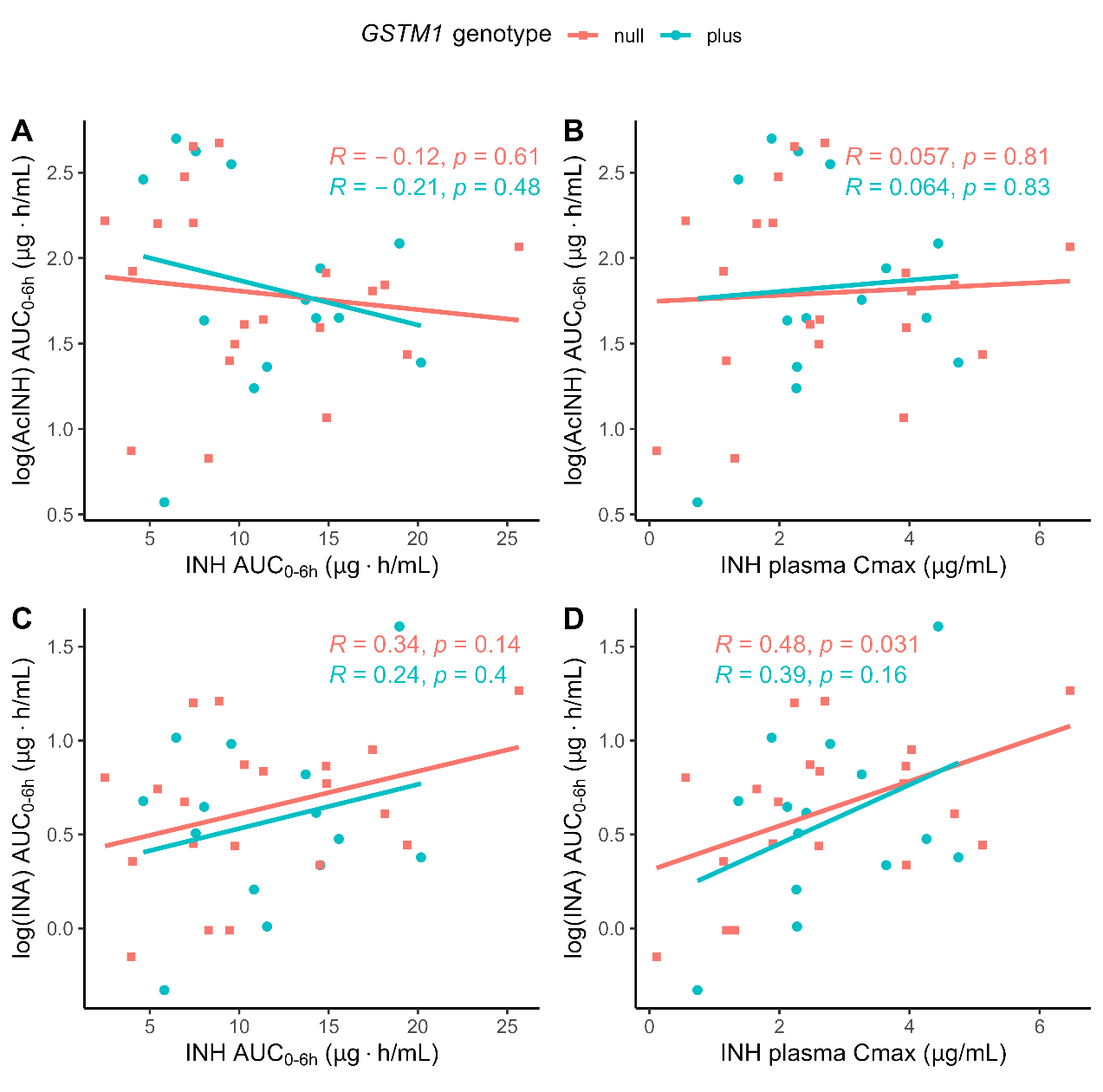


# Supplementary Figure 4. XY plots showing correlation analysis results for isoniazid and two metabolites in blood plasma of patients with tuberculosis. Patient samples were stratified based on the GSTM1 genotype.

(A) Acetylisoniazid AUC_0-6h_ (µg·h /mL) versus isoniazid AUC_0-6h_ (µg·h /mL). (B) Acetylisoniazid AUC_0-6h_ (µg·h /mL) versus isoniazid C_max_ (2 h, µg/mL). (C) Isonicotinic acid AUC_0-6h_ (µg·h /mL) versus isoniazid AUC_0-6h_ (µg·h /mL). (D) Isonicotinic acid AUC_0-6h_ (µg·h /mL) versus isoniazid C_max_ (2 h, µg/mL).

AcINH and INA AUC_0-6h_ values were log-trans formed before running the tests. The solid lines represent the Pearson correlation coefficient. Dots represent the observed data for a single patient.

Abbreviations: INH, isoniazid; AcINH, acetylisoniazid; INA, izonicotinic acid; AUC, area under the concentration-time curve; C_max_, maximum concentration; GSTM1, glutathione S-transferase mu 1 class.
